# Comparison of Prokaryotic and Eukaryotic Communities in Soil Samples with and without Tomato Bacterial Wilt Collected from Different Fields

**DOI:** 10.1264/jsme2.ME17131

**Published:** 2017-11-28

**Authors:** Chol Gyu Lee, Toshiya Iida, Yohei Uwagaki, Yoko Otani, Kazuhiro Nakaho, Moriya Ohkuma

**Affiliations:** 1 Japan Collection of Microorganisms, RIKEN BioResource Center Tsukuba, Ibaraki, 305–0074 Japan; 2 Ishikawa Agriculture and Forestry Research Center Kanazawa, Ishikawa, 920–3198 Japan; 3 Wakayama Agricultural Experiment Station Koinokawa, Wakayama, 640–0423 Japan; 4 Institute of Vegetable and Floriculture Science, National Agriculture and Food Research Organization Tsu, Mie 514–2392 Japan

**Keywords:** 454 pyrosequencing, biocontrol agent, deep layer of soil, indicator species analysis, *Ralstonia solanacearum*

## Abstract

Biocontrol agents (BCA) effectively suppress soil-borne disease symptoms using natural antagonistic prokaryotes or eukaryotes. The main issue associated with the application of BCA is that disease reduction effects are unstable under different field conditions. In order to identify potentially effective BCA among several fields, we compared prokaryotic and eukaryotic communities in soil with and without tomato bacterial wilt from three different fields, each of which had the same field management and similar soil characteristics. Soil samples were collected from three fields and two depths because bacterial wilt pathogens were present in soil at a depth greater than 40 cm. We classified soil samples based on the presence or absence of the bacterial *phcA* gene, a key gene for bacterial wilt pathogenicity and tomato disease symptoms. Pyrosequencing of the prokaryotic 16S rRNA gene and eukaryotic internal transcribed spacer region sequences showed that the diversity and richness of the communities mostly did not correlate with disease symptoms. Prokaryotic and eukaryotic community structures were affected more by regional differences than the appearance of disease. Several prokaryotes and eukaryotes were more abundant in soil that lacked disease symptoms, and eight prokaryotes and one eukaryote of this group were commonly detected among the three fields. Some of these taxa were not previously found in disease-suppressive soil. Our results suggest that several prokaryotes and eukaryotes control plant disease symptoms.

Tomato (*Solanum lycopersicum*) is one of the most important vegetables in the world, with a global annual yield of approximately 160 million tons (FAO. Statistical database [FAOSTAT], 2014; Available from: http://fatstat.fao.org.). Its production is often prevented by bacterial wilt, a devastating disease caused by *Ralstonia solanacearum*. This soil-borne pathogen infects more than 200 plant species, *e.g.*, olive, tomato, tobacco, and eggplant, and, thus, causes great losses in agriculture and horticulture (22). Bacterial wilt disease is principally managed by soil fumigation, resistant cultivars, soil amendments, crop rotation, and field sanitation (47). Biocontrol agents (BCA), which control the bacterial wilt pathogen using microbes, have recently been developed (11, 17, 48). One of the serious issues associated with the application of BCA techniques is that their effects are still hindered by soil types, sampling sites, climate conditions, and other factors (12). Therefore, the control of plant disease by BCA is generally site specific (31).

Soils with high antagonist potential lead to the suppression of soil-borne pathogens. These soils, called disease-suppressive soils, have been reported for multiple soil-borne pathogens including those causing *Fusarium* wilt (29), potato common scab (37), damping-off disease (18), tobacco black root rot (24), and bacterial wilt (41). Soil microbes play important roles in the suppression of plant disease (15, 33, 34). However, the isolation of effective BCA is technically difficult because more than 90% of soil microbes are uncultivable (45). Yin *et al.* compared bacterial communities in soil that suppressed and conduced *Rhizoctonia solani* and found effective bacteria for disease control (49). Therefore, a comparison of microbial communities in disease-suppressive and disease-conducive soil may provide insights for identifying BCA for the soil environment. In our previous study, we also compared prokaryotic communities in soil with and without signs of tomato bacterial wilt symptoms, but with pathogens present, and some prokaryotes were specifically detected in soil that did not demonstrate wilt symptoms (27). However, in previous studies, soil samples were only collected from one field. If microbes that are associated with plant disease suppression are detected among several different fields, they may widely control soil-borne pathogens, instead of only at a specific site.

This study aims to reveal differences in microbial communities in soil samples with and without tomato bacterial wilt symptoms collected from three different sampling sites. Although BCA against the bacterial wilt pathogen have been shown with not only bacterial species, but also several eukaryotic species, investigations on eukaryotic communities in disease-suppressive soil are often ignored (14, 35). Prokaryotes in soil at a depth greater than 40 cm are important because *R. solanacearum* is distributed not only in the plow layer of soil, but also in the hardpan layer (13). We previously revealed that prokaryotic communities differed between soil at depths less than and greater than 40 cm and successfully detected unique prokaryotes at depths greater than 40 cm (27). Therefore, we collected soil samples at two depths, less than and greater than 40 cm, in each field and performed high-throughput sequencing of prokaryotic 16S rRNA genes and eukaryotic internal transcribed spacer (ITS) regions. Each of the fields that we selected to sample were managed identically, but showed the presence and absence of wilt disease. We attempted to find microbes that were specifically detected in soil without disease symptoms among different sampling fields and these specific microbial taxa are BCA candidates to commonly treat bacterial wilt in different regions.

## Materials and Methods

### Sampling sites, soil sampling, and DNA extraction

Soil samples were collected from greenhouses of tomato plants located in Hakusan (Ha-field) and Kahoku City (Ka-field), Ishikawa Prefecture and in Inami city, Wakayama Prefecture (Wa-field) in late January 2015. Detailed information on the sampling sites is shown in Table 1. In the Ka- and Ha-fields, greenhouses with (Ha-1 and Ka-1) and without (Ha-2 and Ka-2) disease symptoms adjoined each other. Bacterial wilt symptoms had not appeared in the Ha-2 and Ka-2 fields for at least five years. In Wa-field, one field showed disease symptoms (Wa-1), whereas two did not (Wa-2 and Wa-3), and they were spatially separated. No significant differences were observed in soil types, field management, or soil chemical properties such as pH and electron conductivity (EC) between soil sites that had or lacked bacterial wilt symptoms in each field (*P*<0.05, *t*-test). No tomato plants were planted in each field at the sampling time. We collected soil samples from two different depths (20–30 and 40–50 cm) in each field using a core sampler (Gauge Auger DIK-106B; Daiki Rika Kogyo, Saitama, Japan). Five samples were collected from each greenhouse in the Ha- and Ka-fields (a total of 40 samples). In Wa-field, five samples were collected from Wa-1 and Wa-3, and three samples were collected from Wa-2 (a total of 26 samples). Sixty-six samples were collected from all fields and stored at −20°C until used. DNA was extracted from 0.5 g of each soil with an ISOIL for the Beads Beating kit (Nippongene, Tokyo, Japan) following the manufacturer’s instructions. The quantity and purity of DNA was measured using a NanoDrop spectrophotometer (Thermo Fischer Scientific, Waltham, MA, USA) and by visualization on a 0.8% agarose gel in Tris-acetate-EDTA (TAE) buffer.

### Assessment of the presence of *R. solanacearum*

The *phcA* gene, which plays a major role in the regulation of *R. solanacearum* pathogenicity (38), was amplified from DNA that was extracted from each soil sample as described by Lee *et al.* (27). Briefly, a two-step nested polymerase chain reaction (PCR) was performed to detect the *phcA* gene; two primer sets were used for the first step (phcA2981f [5′-TGGATATCGGGCTGGCAA-3′] and phcA4741r [5′-CGCTTTTGCGCAAAGGGA-3′]) and for the second step (phcA3538f [5′-GTGCCACAGCATGTTCAGG-3′] and phcA4209r [5′-CCTAAAGCGCTTGAGCTCG-3′]) (19). Amplification was verified by gel electrophoresis (1.5% agarose in TAE buffer). We classified the three soil types based on *phcA* gene detection and pathogenesis, according to Lee *et al.* (27): soil not showing signs of disease, but with *phcA* (S-soil), soil with diseased tomato plants and *phcA* (C-soil), and soil not showing bacterial wilt without *phcA* (unclassified soil).

### Amplification of the prokaryotic 16S rRNA gene and eukaryotic ITS region and tag-encoded amplicon pyrosequencing

PCR was performed on each sample to amplify the V4 variable region of the 16S rRNA gene using the bacterial and archaeal universal primers 515F (5′-GTGCCAGCMGCCGCGGTAA-3′) and 806R (5′-GGAC-TACVSGGGTATCTAA-3′) (4), and the fungal ITS2 region was amplified using ITS3_KYO2 (5′-GATGAAGAAC GYAGYRAA-3′) and ITS4 (5′-TCCTCCGCTTATTGATATGC-3′) coupled with the Roche 454 Titanium sequencing adapters (44). The 515F and ITS3_KYO2 primers contained the barcode sequences with a Roche 454-A pyrosequencing adapter (Titanium Lib-L adapters), and the 806R and ITS4 primers had a Roche 454-B adapter. When two or more bands were detected with 1.5% agarose gel electrophoresis, PCR products of approximately 350 bp in length were excised from the gel and purified using a MonoFas DNA purification kit (GL Sciences, Tokyo, Japan) for prokaryotes and eukaryotes. Eleven soil samples were not amplified with the primers for eukaryotes. Each PCR amplicon was cleaned twice using an Agencourt AMPure XP system (Beckman Coulter, Brea, CA, USA) to remove primers and short DNA fragments and then quantified using a Qubit Fluorometer (Invitrogen, Carlsbad, CA, USA). The purified PCR amplicons were combined in equimolar ratios in a single tube for emulsion PCR (emPCR). An emPCR reaction was performed with an approximate ratio of 0.2:1 (amplicon:emPCR beads), and the amplicon sequencing of 16S rRNA genes and ITS sequences was performed on the Roche 454 GS Junior Titanium sequencer using a Lib-L kit (Roche, Branford, CT, USA). Sequencing data have been deposited in the DNA Data Base of Japan (DDBJ) Sequence Read Archive under accession number DRA005842 and DRA005843 for prokaryotes and eukaryotes, respectively.

### Data analysis

Each raw standard flowgram format (SFF) file was preprocessed in Quantitative Insights Into Microbial Ecology (QIIME) (3). Data from read sequences, quality, flows, and ancillary metadata were analyzed using the QIIME pipeline according to Campisaono *et al.* (2). Quality filtering consisted of discarding reads <200 bp or >500 bp for 16S rRNA and ITS sequences, excluding homopolymer runs of >6 bp and >6 continuous ambiguous bases, but accepting 1 barcode correction and 2 primer mismatches. Moreover, a mean quality score less than 25 was also a criterion used to remove singleton operational taxonomic units (OTUs) and chimeric sequences for statistical analyses. Denoising was performed using the built-in Denoiser algorithm, and chimera removal and OTU picking were accomplished with USEARCH 61 considering a pairwise identity percentage of 0.97. The taxonomy assignment of each OTU was performed using the RDP (Ribosomal database Project) naïve Bayesian classifier against the Greengenes database and GenBank’s Basic Local Alignments Search Tool (BLAST) for prokaryote and eukaryote sequences, respectively. An OTU-based analysis was performed on pyrotag-based datasets to calculate richness and diversity using the phyloseq R package (1.724) (32). The diversity within each individual sample was estimated using the non-parametric Shannon diversity index and Simpson’s diversity index. The Chao1 estimator and observed OTU numbers were calculated to estimate the richness of each sample. A multivariate analysis of community structure and diversity was performed on pyrotag-based datasets using a weighted UniFrac dissimilarity matrix that was calculated in QIIME, jackknifing (1,000 reiterations) read abundance data at the deepest level possible (3,105 and 542 reads for prokaryotes and eukaryotes, respectively), and using unconstrained ordination by a Principal Coordinate Analysis (PCoA) for prokaryotes. Regarding eukaryotes, dissimilarity tests were performed using the Bray-Curtis dissimilarity index between samples (7). Finally, an indicator species analysis was calculated using the indicspecies R package in prokaryotic and eukaryotic communities (9). The indicator species analysis allows for the identification of species that may be used as an indicator of a site group. We created two patterns of soil groups: one group was divided into samples from soil with or without bacterial wilt symptoms, while the other group was divided based on bacterial wilt symptoms in the sample as well as the soil layer. Each result was merged, and abundant OTUs associated with soil without wilt symptoms were evaluated, rather than those from soil with symptoms (*P*<0.001). The number of random permutation tests for the calculation of indicator values was 999.

## Results and Discussion

### Detection of pathogenetic *R. solanacearum*

PCR products of *phcA* were obtained from all fields with disease symptoms, irrespective of soil depth, except for one plot in Wa-field (Wa-1-5) (Table 2). Symptoms associated with *R. solanacearum* were not observed in five, three, and five plots of the Ha-, Ka-, and Wa-fields, respectively; however, the *phcA* gene was detected.

Soils were classified based on *phcA* gene detection and pathogenesis (see Materials and Methods). We selected 5 S-soil and 5 C-soil samples in Ha-field, 3 S-soil and 5 C-soil samples in Ka-field, and 5 S-soil and 4 C-soil samples in Wa-field for further investigation (Table 2). Unclassified soil samples were not investigated further. We did not investigate the Wa-1-5 plot; this plot had tomato plants with disease symptoms, but no *phcA* in the field soil, suggesting that *R. solanacearum* infected above-ground parts of the tomato plant to cause wilt symptoms.

### Diversity and richness in S- and C-soils

Pyrosequencing yielded 402,910 sequences in 54 samples for prokaryotes and 74,226 sequences in 45 samples for eukaryotes (Supplemental Table S1). These sequences were clustered into 8,240 OTUs and 1,369 OTUs for prokaryotes and eukaryotes, respectively. No significant differences were observed in prokaryotic diversity or richness between S-soil and C-soil samples, except for the lower layer of Ha-field (Table 3). In this field, the OTU number and Chao 1 richness estimator were lower in C-soil than in S-soil.

Previous studies indicated that bacterial diversity and richness correlate with the suppression of plant pathogens (11, 29). A study by van Elsas *et al.* revealed a negative correlation between the diversity of soil microbiotas and survival of pathogenic microbes (46). They explained that the underlying mechanisms of diversity-invasiveness relationships may involve competition for the utilization of limited resources. Other studies indicated that the composition of indigenous bacterial populations is simpler in plant disease-conducive than in -suppressive soil (17, 40).

Regarding eukaryotes, Shannon and Simpson’s indices were significantly higher in C-soil than in S-soil for the upper and lower layers in Ka-field (Table 3). OTU numbers were higher in C-soil than in S-soil for the upper layer in Ka-field. However, no significant differences were observed in eukaryotic richness or diversity between S-soil and C-soil. Fungal diversity is higher or lower in soil treated with bio-fertilizer, which suppresses plant disease more than conventional soil, depending on previous studies (25, 30, 36). Fu *et al.* (2017) showed that the application of a bio-fertilizer that suppresses banana *Fusarium* wilt led to greater bacterial richness and diversity, whereas fungal diversity and richness did not appear to correlate with the incidence of this disease (11).

In the present study, no significant differences were observed in the diversity and richness of prokaryotes or eukaryotes between S-soil and C-soil in most fields. Therefore, the relationships among microbial diversity, richness, and disease symptoms remain unclear.

### Microbial community composition and structure in S-soil and C-soil

**Fig. 1:**
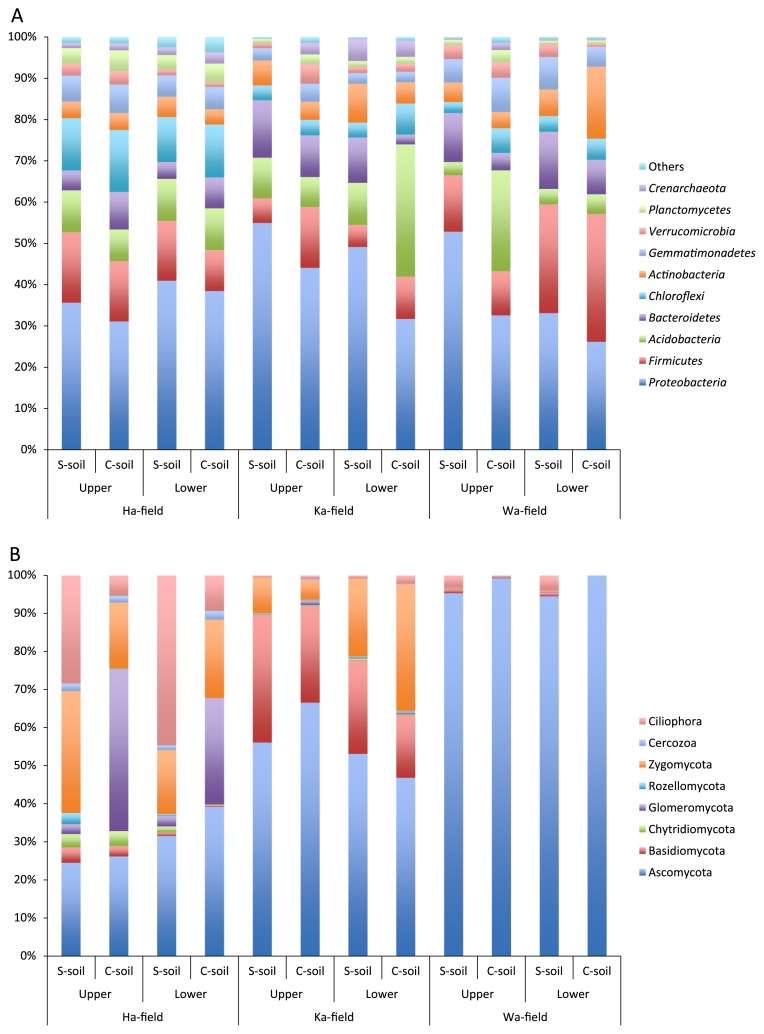
Relative abundance of prokaryotic (A) and eukaryotic (B) phyla in S-soil and C-soil.

Fig. 1 shows the prokaryotic and eukaryotic community compositions of upper- and lower-layer soil samples in the three fields. *Proteobacteria* represented between 26 and 53% of the prokaryotic species in all fields (Fig. 1A, Supplemental Table S2). Their representation was significantly higher in S-soil than in C-soil in both layers of Ha-field and in the lower layer of Ka-field (*t*-test, *P*<0.05). *Firmicutes*, *Acidobacteria*, *Chloroflexi*, and *Bacteroidetes* were the next most dominant prokaryotic phyla in most cases. *Firmicutes* were significantly higher in C-soil in the upper layer of Ka-field, *Acidobacteria* were more abundant in the lower layer of Ka-field and upper layer of Wa-field, and no significant differences were observed in *Chloroflexi* among S-soil and C-soil in all fields.

Regarding eukaryotic communities, Ascomycota accounted for more than 90% of the relative abundance in Wa-field, whereas they occupied approximately 25 to 67% in the Ha- and Ka-fields (Fig. 1B, Supplemental Table S2). Basidiomycota represented 16 to 33% of eukaryotic abundance in Ka-field, and Zygomycota occupied 17 to 33% of eukaryotic abundance in Ha-field and the lower layer of Ka-field. In Ha-field, *Cilliphora* levels were significantly higher in S-soil than in C-soil, while *Glomeromycota* were less abundant in S-soil than in C-soil, irrespective of soil depth. The levels of other phyla were not significantly different between S-soil and C-soil in each field. These results suggest that specified microbes of prokaryotes and eukaryotes in S-soil were not commonly detected among the three fields at the phylum level.

**Fig. 2:**
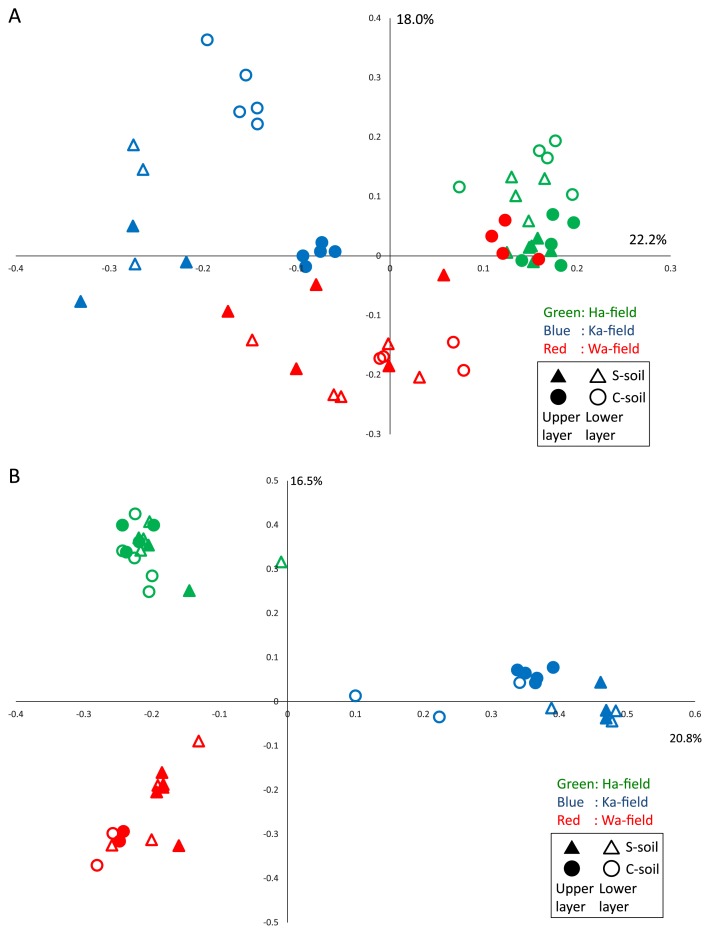
A UniFrac-weighted principal component analysis of prokaryotic (A) and Bray-Curtis principal component analysis of eukaryotic (B) communities in S-soil and C-soil. Closed triangle, S-soil in the upper layer. Open triangle, S-soil in the lower layer. Closed circle, C-soil in the upper layer. Open circle, C-soil in the lower layer. Green plots: Ha-field, Blue plots: Ka-field, Red plots: Wa-field.

PCoAs based on weighted-UniFrac and Bray-Curtis distance analyses showed that prokaryotic and eukaryotic communities were roughly separated along the sampling fields (Fig. 2). Regarding prokaryotes, communities in S-soil were similar with those in C-soil in Ha-field. In Ka-field, communities in S-soil differed from those in C-soil regardless of the soil layer, whereas those in the upper layer only differed in Wa-field (Fig. 2). Communities in C-soil in the upper layer of Wa-field grouped together with those in Ha-field. Similarly, communities in C-soil in the upper layer of Ka-field were closely related to those in Wa-field. We previously compared soil prokaryotic community structures between S-soil and C-soil using the same statistical method (27). The relative abundance of each prokaryotic phylum was not significantly different between S-soil and C-soil, whereas community structures in the upper soil layer were distinctly classified as different between S-soil and C-soil. Soil samples were collected from the same greenhouse; therefore, a difference in the relative abundance of each prokaryote was not detected at the phylum level. Eukaryotic community structures were more clearly differentiated among sampling fields than soil classification based on the signs of bacterial wilt (Fig. 2). Prokaryotic and eukaryotic community structures were affected more by regional differences than the appearance of disease.

### Specific prokaryotes detected in soil without symptoms

A total of 230, 151, and 123 OTUs of prokaryotes were significantly more abundant in S-soil than in C-soil in the Ha-, Ka-, and Wa-fields, respectively (Table 4, Supplemental Table S3). Regarding S-soil-abundant prokaryotic OTUs, *Proteobacteria* were mainly detected in both layers in all three fields. *Bacteroidetes* OTUs were the second most abundant, having higher numbers in the Ka- and Wa-fields, and most of them belonged to *Sphingobacteriia*. *Acidobacteria* OTUs were commonly detected as the second most abundant group in the upper and lower layers of soil in Ha-field, whereas an *Acidobacteria* OTU was not detected in the lower layer of Ka-field or the upper layer of Wa-field.

Nine, fifteen, and twenty-seven OTUs were shared between the Ha- and Ka-fields, Ha- and Wa-fields, and Ka- and Wa-fields, respectively (Table 5). They mainly belonged to *Proteobacteria* (27 OTUs), *Bacteroidetes* (9 OTUs), *Firmicutes* (6 OTUs), and *Actinobacteria* (4 OTUs). Twenty-four out of 27 OTUs that were shared between the Ka- and Wa-fields belonged to *Proteobacteria* or *Bacteroidetes* and were not differentiated by the soil layers. Seven OTUs were commonly detected as significantly abundant OTUs from S-soil in all three sampling fields. Among them, the OTUs of *Sphingobacteriaceae* and *Dokdonella* were commonly detected from the upper and lower soil layers in the Ha- and Ka-fields and in the Ha- and Wa-fields, respectively. *Sphingomonas* spp. belonging to *Sphingomonadaceae* are known to suppress soil-borne diseases and produce plant hormone-like compounds that promote plant growth (23, 24, Yang, H., J. Niu, J. Tao, *et al.* 2016. The impacts of different green manure on soil microbial communities and crop health. Agric Sci Agronomy. Preprint. Available from: 2016090056. doi: 10.20944/preprints201609.0056.v1). However, the abundance of *Dokdonella*-like bacteria has been positively correlated with *Fusarium* disease rates in Chinese fields (17, 28, 39). OTUs in candidate division WPS-1, *Rhodocyclaceae*, and *Aquihabitans* were mainly detected from the upper layer of all fields. *Azoarcus* and *Azospira*, as *Rhodocyclacea* members, are generally involved in nitrogen cycling in soil environments. Bacteria involved in soil nitrogen cycling affect the suppression of plant diseases, and their release of nitrogen influences the microbial community structure (8, 16). An *Aquihabitans* species was recently isolated from a freshwater environment, and its ecology has yet to be examined in detail (21). An OTU in *Gemmatimonas* was detected in the lower layer in all three fields. *Gemmatimonas* members are reported to be more abundant bacteria in suppressive soil for *Fusarium* wilt, and are difficult to isolate due to their slow growth rate (6). An OTU in *Caldicoprobacter* (belonging to *Clostridia*) was detected uniquely as an obligate anaerobic bacterium in all fields, and was frequently detected in the lower soil layer. *Caldicoprobacter* is a spore-forming, non-motile, and xylanolytic bacterium that was isolated from sheep feces (50). Taylor and Guy showed that bacteria related to *Clostridium*, in combination with *Bacillus*, were effective for controlling the pathogenic fungus *Peniophora sacrata* (43).

*Sphingomonadaceae* and *Rhodocyclaceae* were commonly detected in our previous study (27) and from S-soil in the present study. These bacteria have the potential to be effective BCA among several fields. Our previous study also uniquely detected several prokaryotic OTUs from S-soil that may be involved in nitrogen cycling bacteria from S-soil. Moreover, we also commonly found the anaerobic bacteria, *Caldicoprobacter* and unidentified *Anaerilineae* in S-soil in the present and previous studies. They may play important roles in the suppression of bacterial wilt.

### Specific eukaryotes detected in soil without symptoms

Eighty, thirty-nine, and six OTUs of eukaryotes were significantly more abundant in S-soil than in C-soil in the Ha-, Ka-, and Wa-fields, respectively (Table 4, Supplemental Table S4). Among them, the main phylum that was specifically detected in S-soil in all fields was Ascomycota. Basidiomycota was dominant in Ka-field, and members of protists and Viridiplantae were mainly detected in Ha-field. Eleven and nine OTUs were shared between the Ha- and Ka-fields and Ha- and Wa-fields, respectively, whereas one OTU was commonly detected in the Ka- and Wa-fields and another OTU was shared in all fields. Twelve out of 22 OTUs were closely related to Ascomycota. Other OTUs belonged to Basidiomycota (3 OTUs); Mucoromycota (2 OTUs) and unidentified fungi (1 OTU) of the fungal phyla; Ecdysozoa (1 OTU), belonging to Metazoa; and Streptophyta (2 OTU) and Chlorophyta (1 OTU), belonging to Viridiplantae (Table 6). Several OTUs that were abundant in S-soil from more than two fields were identified as BCA in the previous study. *Plectosphaerella*, *Epicoccum*, *Drechslerella*, and *Clonostachys* were identified as BCA for cyst nematodes, the brown rot of peach, reniform nematodes, and damping off of carrot caused by *Alternaria*, respectively (1, 5, 20, 26). In a previous study, *Glomus versiforme*, *Pythium oligandrum*, *Gigaspora margarita*, *G. mosseae, Scutellospora* sp., and the lichen *Parmotrema tinctorum* were identified as BCA against *R. solanacearum*, and *Trichoderma*, *Gliocladium*, *Penicillium*, and *Acremonium* spp. are also well-known BCA species for several plant diseases (45, 51). However, these eukaryotes were not found in the present study. *Fusarium tricinctum* was commonly abundant in S-soil in all fields, and members of the genus of *Fusarium* are well-known plant pathogens (Table 6). *Pratylenchus* members are well-known root-lesion nematodes for banana, and plant parasitic nematodes appear to increase the severity of bacterial wilt (10, 42). However, no disease symptoms were observed on tomato plants in S-soil in the fields in spite of the detection of these fungal pathogens.

## Conclusion

In the present study, we compared prokaryotic and eukaryotic communities in soil that had or lacked bacterial wilt, and commonly detected specific microbes in S-soil in three different fields. Neither soil prokaryotic and eukaryotic diversity nor richness correlated with the presence or absence of bacterial wilt symptoms. Prokaryotic and eukaryotic community structures were affected more by regional differences than the appearance of disease. A total of 504 and 125 OTUs were more abundant in S-soil for prokaryotes and eukaryotes, respectively. Some OTUs were commonly detected in S-soil in more than two fields, and some of them were well-known plant pathogens. However, since the signs of plant disease were not observed in S-soil, several prokaryotes and eukaryotes may control plant disease symptoms. Future studies are needed in order to elucidate the roles and effectiveness of these microbes as well as the relationship between soil suppressiveness and soil microbes or soil chemical and physical properties.

## Supplementary Material



## Figures and Tables

**Table 1:** Detailed information on fields.

Prefecture	Ishikawa				Wakayama		
		
City	Hakusan		Kahoku		Inami		
			
Field name	Ha-1	Ha-2	Ka-1	Ka-2	Wa-1	Wa-2	Wa-3
Latitude and longitude	36° 29′ N, 136° 30′ E		36° 45′ N, 136° 45′ E		33° 27′ N, 135° 14′ E	33° 49′ N, 135° 15′ E	33° 38′ N, 135° 14′ E
Soil types	Gleysol		Fluvisol		Cambisols	Cambisols	Cambisols
Pathogenesis	+	−	+	−	+	−	−
Sample number	5	5	5	5	5	3	5
pH	7.22±0.19	7.02±0.14	5.67±0.14	5.51±0.27	7.49±0.33	7.39±014	7.08±047
EC (mS cm^−1^)	0.31±0.07	0.31±0.03	0.31±0.22	0.84±0.17	0.88±0.29	0.19±005	0.21±006

**Table 2:** Pathogenesis and *phcA* amplification of each soil sample.

Plot name	Sample number	Soil layer	*phcA* gene	Disease	Classification*
Ha-1	1	Up	++	−	S-soil
		Low	++	−	
	2	Up		−	
		Low	++	−	
	3	Up	++	−	
		Low	+	−	
	4	Up		−	
		Low	++	−	
	5	Up	++	−	
		Low	+++	−	

Ha-2	1	Up	++	+	C-soil
		Low	+	+	
	2	Up	+	+	
		Low	++	+	
	3	Up		+	
		Low	+	+	
	4	Up		+	
		Low	+++	+	
	5	Up		+	
		Low	++	+	

Ka-1	1	Up		−	Unclassified soil
		Low		−	
	2	Up		−	
		Low		−	

	3	Up		−	S-soil
		Low	+	−	
	4	Up		−	
		Low	+	−	
	5	Up		−	
		Low	+	−	

Ka-2	1	Up		+	C-soil
		Low	+	+	
	2	Up		+	
		Low	++	+	
	3	Up		+	
		Low	+	+	
	4	Up		+	
		Low	+++	+	
	5	Up		+	
		Low	+	+	

Wa-1	1	Up	+	+	C-soil
		Low	+	+	
	2	Up	+	+	
		Low	+	+	
	3	Up	+	+	
		Low		+	
	4	Up		+	
		Low	+	+	

	5	Up		+	Unclassified soil
		Low		+	

Wa-2	1	Up		−	
		Low		−	
	2	Up		−	
		Low		−	

	3	Up		−	S-soil
		Low	+	−	

Wa-3	1	Up	+	−	S-soil
		Low	++	−	
	2	Up	+	−	
		Low	+	−	
	3	Up	++	−	
		Low	+	−	
	4	Up	+	−	
		Low		−	

	5	Up		−	Unclassified soil
		Low		−	

* For classification, see the text. The intensity of each band was represented by +, ++, and +++, as a weak, moderate, and strong band appearance, respectively.

**Table 3:** Comparison of prokaryotic and eukaryotic diversity and richness indices in S-soil and C-soil in each field

			Ha-field		Ka-field		Wa-field	
		
			S-soil	C-soil	S-soil	C-soil	S-soil	C-soil
Prokaryotes	Shannon	Upper	0.99±0.001	0.99±0.001	0.98±0.03	0.99±0.001	0.84±0.2	0.97±0.02
		Lower	0.99±0.01	0.99±0.001	0.99±0.01	0.97±0.01	0.97±0.02	0.94±0.05
	Simpson	Upper	5.87±0.07	5.96±0.04	5.35±0.77	5.7±0.13	4.24±1.65	5.12±0.27
		Lower	5.82±0.22	5.62±0.15	5.65±0.3	5.01±0.26	5.06±0.28	4.35±0.7
	OTU number	Upper	671±28	658±22	586±145	659±47	406±179	471±36
		Lower	675±48*	575±44	636±60	517±96	471±25	348±65
	Chao1	Upper	1287±57	1195±66	1268±306	1390±111	704±299	798±56
		Lower	1308±85*	1061±69	1314±126	1093±202	840±91	614±123

Eukaryotes	Shannon	Upper	2.93±0.63	2.91±0.53	2.81±0.14	3.73±0.1*	2.27±0.39	1.81±0.03
		Lower	3.17±0.64	2.13±0.77	2.83±0.09	3.57±0.32*	2.41±1.07	2.14±0.12
	Simpson	Upper	0.84±0.13	0.85±0.07	0.87±0.02	0.93±0.01*	0.77±0.06	0.66±0.09
		Lower	0.89±0.05	0.71±0.23	0.85±0.03	0.94±0.03*	0.74±0.25	0.81±0.02
	OTU	Upper	104±23	104±12	170±32	265±40*	68±32	53±17
		Lower	164±129	63±12	178±23	172±46	72±36	32±25
	Chao1	Upper	122±32	113±25	189±43	261±27	90±34	63±15
		Lower	143±79	84±40	193±36	223±26	97±56	65±16

Asterisks represent a pair of means with significantly higher values than those of the other soil type in each field (t-test, *P*<0.05).

**Table 4:** OTU numbers of prokaryotic and eukaryotic phyla that are significantly abundant in S-soil in each field

	Domain	Phylum	Ha-field			Ka-field			Wa-field		
		
			All	Upper	Lower	All	Upper	Lower	All	Upper	Lower
Prokaryotes	Bacteria	*Proteobacteria*	22	21	25	21	10	5	13	5	4
		*Bacteroidetes*	3	4	6	16	3	6	16	3	4
		*Acidobacteria*	15	4	23	11	2	0	2	0	2
		*Firmicutes*	9	10	11	7	0	4	2	2	2
		*Actinobacteria*	4	2	7	11	1	2	2	0	2
		*Chloroflexi*	6	5	6	0	0	1	0	1	1
		*Planctomycetes*	4	2	5	1	0	0	0	0	2
		*Verrucomicrobia*	2	1	3	2	0	0	4	2	0
		*Gemmatimonadetes*	1	1	3	0	0	1	2	0	2
		*Chlamydiae*	3	3	2	0	0	0	0	1	0
		Others	9	6	13	0	2	3	0	1	1

Eukaryotes	Fungi	Ascomycota	4	0	18	8	2	2	2	0	3
		Basidiomycota	1	1	2	11	1	2	0	1	0
		Mucoromycota	0	1	1	5	1	1	0	0	0
		Zoopagomycota	0	0	0	1	0	0	0	0	0
		Unidentified Fungi	0	1	1	0	1	1	0	0	0

	Protists	Ciliophora	5	0	15	0	1	0	0	0	0
		Cercozoa	0	0	3	0	0	0	0	0	0
		Adenophorea	1	0	1	0	0	0	0	0	0

	Viridiplantae	Streptophyta	6	0	8	0	0	0	0	0	0
		Chlorophyta	4	0	7	2	0	0	0	0	0

**Table 5:** Shared prokaryote OTUs that are significantly abundant in S-soil in different fields

Detected field	OTU number	Ha-field		Ka-field		Wa-field		Phylum	Class	Closest relatives
		
		Up	Low	Up	Low	Up	Low			
All	OTU8949	+	+	+		+		candidate division WPS-1		
	OTU2469	+	+	+		+		*Proteobacteria*	*Betaproteobacteria*	*Rhodocyclaceae*
	OTU17222	+		+		+	+	*Actinobacteria*	*Actinobacteria*	*Aquihabitans*
	OTU6877	+	+	+	+		+	*Proteobacteria*	*Alphaproteobacteria*	*Sphingomonadaceae*
	OTU5062	+	+		+	+	+	*Proteobacteria*	*Betaproteobacteria*	*Dokdonella*
	OTU424		+	+	+		+	*Gemmatimonadetes*	*Gemmatimonas*	*Gemmatimonas*
	OTU411		+	+			+	*Firmicutes*	*Clostridia*	*Caldicoprobacter*

Ha & Ka	OTU24779	+		+	+			*Actinobacteria*	*Actinobacteria*	*Streptosporangium*
	OTU15276	+		+	+			*Firmicutes*	*Bacilli*	*Cohnella*
	OTU19407	+	+		+			*Acidobacteria*	*Acidobacteria_Gp6*	*Acidobacteria_Gp6*
	OTU13095	+	+		+			*Chlamydiae*	*Chlamydiia*	*Chlamydiales*
	OTU10784	+	+		+			candidate division WPS-1		
	OTU14695		+	+	+			*Proteobacteria*	*Alphaproteobacteria*	*Phenylobacterium*
	OTU18851		+	+	+			*Proteobacteria*	*Gammaproteobacteria*	*Gammaproteobacteria*
	OTU20848	+			+			*Firmicutes*	*Clostridia*	*Romboutsia*
	OTU14704	+			+			*Proteobacteria*	*Alphaproteobacteria*	*Rhodospirillales*

Ha & Wa	OTU22759	+	+			+	+	*Firmicutes*	*Clostridia*	*Lachnospiraceae*
	OTU24142	+	+			+	+	*Proteobacteria*	*Alphaproteobacteria*	*Alphaproteobacteria*
	OTU170	+	+			+	+	*Verrucomicrobia*	Subdivision3	
	OTU15704	+				+		*Planctomycetes*	*Planctomycetaceae*	*Planctomycetaceae*
	OTU5215	+	+				+	*Actinobacteria*	*Actinobacteria*	*Micromonosporaceae*
	OTU14625	+	+				+	*Bacteroidetes*		
	OTU21179	+	+				+	*Firmicutes*	*Clostridia*	*Clostridium*
	OTU8063		+			+	+	*Bacteroidetes*	*Sphingobacteriia*	*Chitinophagaceae*
	OTU18274		+			+	+	*Gemmatimonadetes*	*Gemmatimonadetes*	*Gemmatimonas*
	OTU20912		+			+	+	*Proteobacteria*	*Alphaproteobacteria*	*Altererythrobacter*
	OTU11041		+			+	+	*Proteobacteria*	*Betaproteobacteria*	
	OTU12798		+				+	*Verrucomicrobia*	Subdivision3	
	OTU23592	+					+	*Candidatus Saccharibacteria*		
	OTU14570	+					+	*Firmicutes*	*Bacilli*	*Bacillales*
	OTU13239	+					+	*Planctomycetes*	*Planctomycetia*	*Pirellula*

Ka & Wa	OTU18820			+	+	+	+	*Bacteroidetes*	*Sphingobacteriia*	*Pedobacter*
	OTU13685			+	+	+	+	*Bacteroidetes*	*Sphingobacteriia*	*Flavisolibacter*
	OTU450			+	+	+	+	*Bacteroidetes*	*Flavobacteriia*	*Flavobacterium*
	OTU7089			+	+	+	+	*Proteobacteria*	*Alphaproteobacteria*	*Sphingomonas*
	OTU16801			+	+	+	+	*Proteobacteria*	*Gammaproteobacteria*	*Rheinheimera*
	OTU10791			+	+	+	+	*Proteobacteria*	*Betaproteobacteria*	*Achromobacter*
	OTU15731			+	+	+	+	*Verrucomicrobia*	*Opitutae*	*Opitutus*
	OTU10170			+	+	+		*Proteobacteria*	*Gammaproteobacteria*	*Gammaproteobacteria*
	OTU19779			+	+	+		*Proteobacteria*	*Alphaproteobacteria*	*Devosia*
	OTU24775			+		+	+	*Bacteroidetes*	*Flavobacteriia*	*Ohtaekwangia*
	OTU7082			+		+	+	*Proteobacteria*	*Alphaproteobacteria*	*Bradyrhizobiaceae*
	OTU1098			+		+	+	*Proteobacteria*	*Betaproteobacteria*	*Betaproteobacteria*
	OTU12616			+		+		*Proteobacteria*	*Alphaproteobacteria*	*Alphaproteobacteria*
	OTU52			+	+		+	*Bacteroidetes*	*Flavobacteriia*	*Flavobacterium*
	OTU3636			+	+		+	*Proteobacteria*	*Alphaproteobacteria*	*Devosia*
	OTU1963				+	+	+	*Bacteroidetes*	*Sphingobacteriia*	*Flavisolibacter*
	OTU19716				+	+	+	*Bacteroidetes*	*Sphingobacteriia*	*Terrimonas*
	OTU8299				+	+	+	*Proteobacteria*	*Betaproteobacteria*	*Cupriavidus*
	OTU3281				+	+	+	*Proteobacteria*	*Gammaproteobacteria*	*Pseudohaliea*
	OTU10190				+	+	+	*Proteobacteria*	*Alphaproteobacteria*	*Bosea*
	OTU21838				+	+	+	*Proteobacteria*	*Alphaproteobacteria*	*Dongia*
	OTU22451				+	+	+	*Proteobacteria*	*Alphaproteobacteria*	*Rhizomicrobium*
	OTU16175				+	+	+	*Proteobacteria*	*Gammaproteobacteria*	*Rhodanobacter*
	OTU16117				+	+	+	*Proteobacteria*	*Betaproteobacteria*	*Ramlibacter*
	OTU13358				+	+	+	*Proteobacteria*	*Alphaproteobacteria*	*Ensifer*
	OTU7168				+		+	*Actinobacteria*	*Actinobacteria*	*Pseudonocardineae*
	OTU18310			+			+	*Acidobacteria*	*Acidobacteria_Gp3*	*Acidobacteria_Gp3*

**Table 6:** Shared eukaryote OTUs that are significantly abundant in S-soil in different fields.

Detected field	OTU name	Ha-field		Ka-field		Wa-field		Kingdom	Division	Closest relatives
		
		Up	Low	Up	Low	Up	Low			
All	OTU28		+	+			+	Fungi	Ascomycota	*Fusarium tricinctum*

Ha & Ka	OTU40	+	+	+				Viridiplantae	Chlorophyta	*Chlamydomonas sphagnophila*
	OTU989		+	+	+			Fungi	Ascomycota	*Sporendonema purpurascens* strain KACC 41227
	OTU514		+	+	+			Fungi	Basidiomycota	*Trichosporon* sp. ‘shinodae’ culture-collection CBS:9979
	OTU307		+	+	+			Fungi	Basidiomycota	*Trichosporon* sp. ‘shinodae’ culture-collection CBS:9979
	OTU286		+	+	+			Fungi	Mucoromycota	*Umbelopsis vinacea* strain CBS 236.82
	OTU882		+	+	+			Fungi	Mucoromycota	*Cunninghamella elegans* isolate CFR-C10
	OTU267		+	+				Fungi	Ascomycota	*Pseudospiromastix tentaculata* CBS 184.92
	OTU505		+	+				Fungi	Ascomycota	*Epicoccum nigrum* strain Zbf-S21
	OTU437		+	+				Fungi	Ascomycota	*Zopfiella*sp. TMS-2011 voucher BGd10p15-14
	OTU278		+		+			Fungi	Basidiomycota	*Coprinopsis semitalis* strain CBS291.77
	OTU298		+		+			Fungi	Ascomycota	*Drechslerella dactyloides* strain 03CQ1-7

HA & Wa	OTU68	+	+			+	+	Fungi	Ascomycota	*Lecanicillium aphanocladii*
	OTU156	+	+			+	+	Viridiplantae	Streptophyta	*Imbribryum blandum*
	OTU3	+	+				+	Viridiplantae	Streptophyta	*Imbribryum blandum*
	OTU825	+	+				+	Fungi	Unidentified FungiEctomycorrhizal fungi isolate ECM 289	
	OTU795	+					+	Fungi	Ascomycota	*Geomyces sp.* 23WI14 1
	OTU757		+			+	+	Fungi	Ascomycota	*Plectosphaerella cucumerina* isolate Raph-3
	OTU152		+			+	+	Fungi	Ascomycota	*Plectosphaerella cucumerina* isolate Raph-3
	OTU79		+			+	+	Fungi	Ascomycota	*Fusarium* sp. strain SAR19
	OTU680		+				+	Metazoa	Ecdysozoa	*Pratylenchus goodeyi* isolate PgoKL4 clone 4

Ka & Wa	OTU33				+		+	Fungi	Ascomycota	*Clonostachys rosea*

## References

[1] Atkins S.D., Clark I.M., Sosnowska D., Hirsch P.R., Kerry R.R. (2003). Detection and quantification of *Plectosphaerella cucumerina*, a potential biological control agent of potato cyst nematodes, by using conventional PCR, real-time PCR, selective media, and baiting. Appl Environ Microbiol.

[2] Campisaono A., Antonielli L., Pancher M., Yousaf S., Pindo M., Pertot I. (2014). Bacterial endophytic communities in the grapevine depend on pest management. PLoS ONE.

[3] Caporaso J.G., Kuczynski J., Stombaugh J. (2010). QIIME allows analysis of high-throughput community sequencing data. Nat Methods.

[4] Caporaso J.G., Lauber C.L., Walters W.A., Berg-Lyons D., Lozupone C.A., Turnbaugh P.J., Fierer N., Knight R. (2011). Global patterns of 16S rRNA diversity at a depth of millions of sequence per sample. Proc Natl Acad Sci USA.

[5] Castillo J.D., Lawrence K.S., Klopper J.W., van Santen E. (2010). Evaluation of *Drechslerella dactyloides*, *Drechslerella brochopaga*, and *Paecilomyves Lilacinus* for biocontrol of *Rotylenchulus reniformis*. Nematropica.

[6] Cha J.Y., Han S., Hong H.J. (2016). Microbial and biochemical basis of Fusarium wilt-suppressive soil. ISME J.

[7] Clarke K.R. (1993). Non-parametric multivariate analysis changes in community structure. Aust J Ecol.

[8] Cohen M.F., Yamasaki H., Mazzola M. (2005). *Brassica napus* seed meal soil amendment modifies microbial community structure, nitric oxide production and incidence of Rhizoctonia root rot. Soil Biol Biochem.

[9] De Cáceres M., Legendre P. (2009). Associations between species and groups of sites: indices and statistical inference. Ecology.

[10] Deberdt P., Quénéhervé P., Darrasse A., Prior P. (1999). Increased susceptibility to bacterial wilt in tomatoes by nematode galling and the role of the *Mi* gene in resistance to nematodes and bacterial wilt. Plant Pathol.

[11] Fu L., Penton C.R., Ruan Y., Shen Z., Xue C., Li R., Shen Q. (2017). Inducing the rhizosphere microbiome by biofertilizer application to suppress banana Fusarium wilt disease. Soil Biol Biochem.

[12] Goswami B.K., Pandey R.K., Singh S., Rathour K.S., Ahamad S. (2009). Biopesticides: an ecolofriendly, sustainable and cost effective approach for integrated disease and insect pest management of agricultural crops. Plant Disease Management for Sustainable Agriculture.

[13] Graham J., Lloyd A.B. (1979). Survival of potato strain (race 3) of *Pseudomonas solanacearum* in the deeper soil layers. Aust J Agric Res.

[14] Hase S., Shimizu A., Nakaho K., Takenaka S., Takahashi H. (2006). Induction of transient ethylene and reduction in severity of tomato bacterial wilt by *Pythium oligandrum*. Plant Pathol.

[15] Ho W.C., Cherm L.L., Ko W.H. (1988). *Pseudomonas solanacearum*-suppressive soils in Taiwan. Soil Biol Biochem.

[16] Hossain S., Bergkvist G., Glinwood R., Berglund K., Måretensson A., Hallin S., Persson P. (2015). Brassicaceae cover crops reduce Aphanomyces pea root rot without suppressing genetic potential of microbial nitrogen cycling. Plant Soil.

[17] Huang J., Wei Z., Tan S., Mei X., Yin S., Shen Q., Xu Y. (2013). The rhizosphere soil of diseased tomato plants as a source for novel microorganisms to control bacterial wilt. Appl Soil Ecol.

[18] Hunter P.J., Petch G.M., Calvo-Bado L.A., Pettitt T.R., Parsons N.R., Morgan J.A., Whipps J.M. (2006). Difference in microbial activity and microbial populations of peat associated with suppression of dumping-off disease caused by *Pythium sylvaticum*. Appl Environ Microbiol.

[19] Inoue Y., Nakaho K. (2014). Sensitive quantitative detection of *Ralstonia solanacearum* in soil by the most probable number-polymerase chain reaction (MPN-PCR) method. Appl Microbiol Biotechnol.

[20] Jensen B., Knudsen I.M.B., Madsen M., Jensen D.F. (2004). Biopriming of infected carrot seed with an antagonist, *Clonostachys rosea*, selected for control of seedborne *Alternaria* spp. Phytopathol.

[21] Jin L., Huy H., Kim K.K. (2013). *Aquihabitans darchungensis* gen nov, sp nov, an actinobacterium isolated from reservoir water. Int J Sys Evol Microbiol.

[22] Kelman A., Prior P.H., Allen C., Elphinstone J. (1998). One hundred and one years of research on bacterial wilt. Bacterial Wilt Disease: Molecular and Ecological Aspects.

[23] Khan A.L., Waqas M., Kang S.M. (2014). Bacterial endophyte *Sphingomonas* sp LK11 produces giberellines and IAA and promotes plant growth. J Microbiol.

[24] Kyselková M., Kopecky J., Frapolli M., Défago G., Ságová-Marecková M., Grundmann G.L., Moënne-Loccoz Y. (2009). Comparison of rhizobacterial community composition in soil suppressive or conductive to tobacco black root rot disease. ISME J.

[25] Lang J., Hu J., Ran W., Xu Y., Shen Q. (2012). Control of cotton *Verticillium* wilt nad fungal diversity of rhizosphere soils by bio-organic fertilizer. Biol Fertil Soils.

[26] Larena I., Torres R., De Cal A. (2005). Biological control of postharvest brown rot (*Monilinia* spp) of peaches by field applications of Epicoccum nigrum. Biol Control.

[27] Lee C.G., Iida T., Inoue Y., Muramoto Y., Watanabe H., Nakaho K., Ohkuma M. (2017). Prokaryotic Communities at different depths between soils with and without tomato bacterial wilt but pathogen-present in a single greenhouse. Microbes Environ.

[28] Li R., Shen Z., Sun L., Zhang R., Fu L., Deng X., Shen Q. (2016). Novel soil fumigation method for suppressing cucumber *Fusarium* wilt disease associated with soil microflora alterations. Appl Soil Ecol.

[29] Li X., Zhang Y., Ding C., Jia Z., He Z., Zhang T., Wang X. (2015). Declined soil suppressiveness to *Fusarium oxysporum* by rhizosphere microflora of cotton in soil sickness. Biol Fertil Soils.

[30] Liu Y., Shi J., Feng Y., Yang X., Li X., Shen Q. (2013). Tobacco bacterial wilt can be biological controlled by the application of antagonistic strains in combination with organic fertilizer. Biol Fertil Soils.

[31] Malusà E., Pinzari F., Canfora L., Singh D.P., Singh H.D., Prabha R. (2016). Efficacy of biofertilizers: challenges to improve crop production. Microbial Inoculants in Sustainable Agricultural Productivity, vol 2 Functional Applications.

[32] McMurdie P.J., Holmes S. (2013). phyloseq: An R package for reproducible interactive analysis and graphics of microbiome census data. PLoS ONE.

[33] Mendes R., Kruijt M., de Bruijin I. (2011). Deciphering the rhizosphere microbiome for disease-suppressive bacteria. Science.

[34] Nishiyama M., Shiomi Y., Suzuki S., Marumoto T. (1999). Suppression of growth of *Ralstonia solanacearum*, tomato bacterial wilt agent, on/in tomato seedlings cultivated in a suppressive soil. Soil Sci Plant Nutr.

[35] Pacumbaba R.P., Beyl C.A., Pacumbaba R.O. (1999). Shiitake Mycelial leachate suppresses growth of some bacterial species and symptoms of bacterial wilt of tomato and lima bean *in vitro*. Plant Dis.

[36] Qiu M., Zhang R., Xue C., Zhang A., Li S., Zhang N., Shen Q. (2012). Application of bio-organic fertilizer can control *Fusarium* wilt of cucumber plants by regulating microbial community of rhizosphere soil. Biol Fertil Soils.

[37] Rosenzweig N., Tiedje J.M., Quensen J.F., Meng Q., Hao J.J. (2012). Microbial communities associated with potato common scab-suppressive soil determined by pyrosequencing analyses. Plant Dis.

[38] Schell M.A. (2000). Control of virlence and pathogenicity genes of *Ralstonia solanacearum* by an elaborate sensory network. Annu Rev Phytophathol.

[39] Shen Z., Ruan Y., Chao X., Zhang J., Li R., Shen Q. (2015). Rhizosphere microbial community manipulated by 2 years of consecutive biofertilizer application associated with banana *Fusarium* wilt disease suppression. Biol Fertil Soils.

[40] Shen Z., Ruan Y., Xue C., Zhong S., Li R., Shen Q. (2015). Soils naturally suppressive to banana *Fusarium* wilt disease harbor unique bacterial communities. Plant Soil.

[41] Shiomi Y., Nishiyama M., Onizuka T., Marumoto T. (1999). Comparison of bacterial community structure in the rhizosplane of tomato plants grown in soils suppressive and conductive towards bacterial wilt. Appl Environ Microbiol.

[42] Talwana H.A., Sprijer P.R., Gold C.S., Swennen R.L., Waele D.D. (2003). A comparison of the effects of the nematodes *Radopholus simils* and *Pratylenchus goodeyi* on growth, root health and yield of an East African highland cooking banana (Musa AAA group). Int J Pest Manage.

[43] Taylor J.B., Guy E.M. (1981). Biological control of root-infecting *Basidomycetes* by species of *Bacillus* and *Clostridium*. New Phytol.

[44] Toju H., Tanabe A.S., Yamamoto S., Sato H. (2012). High-coverage ITS primers for the DNA based identification of Ascomycetes and Basidiomycetes in environmental samples. PLoS ONE.

[45] van Bruggen A.H.C., Semenov A.M. (2000). In search of biological indicators for soil health and disease suppression. Appl Soil Ecol.

[46] van Elsas J.D., Chiurazzi A., Mallon C.A., Elhottovã D., Krištufek V., Flacão J. (2012). Microbial diversity determines the invasion of soil by bacterial pathogen. Proc Natl Acad Sci USA.

[47] Wang J.F., Lin C.H. (2005). Integrated management of tomato bacterial wilt.

[48] Wu B., Wang X., Yang L. (2016). Effects of *Bacillus amyloliquefaciens* ZM9 on bacterial wilt and rhizosphere microbial communities of tobacco. Appl Soil Environ.

[49] Yin C., Hulbert S.H., Schroeder K.L., Mavrodi O., Mavrodi D., Dhingra A., Schillinger W.F., Paulitz T.C. (2013). Role of bacterial communities in the natural suppression of *Rhizoctonia solani* bare patch disease of wheat (*Triticum aestivum* L.). Appl Environ Microbiol.

[50] Yokoyama H., Wagner I.D., Wiegei J. (2010). *Caldicoprobacter oshimai* gen nov, sp nov, an anaerobic xylanolytic, extremely thermophilic bacterium isolated from sheep faces, and proposal of Caldicoprobacteraceae fam nov. Int J Sys Evol Microbiol.

[51] Yuliar, Nion Y.A., Toyota K. (2015). Recent trends in control methods for bacterial wilt disease caused by *Ralstonia solanacearum*. Microbes Environ.

