# Supplementary material for: Comparison of Prokaryotic and Eukaryotic Communities in Soil Samples with and without Tomato Bacterial Wilt Collected from Different Fields
Source: Microbes Environ. 2017 Nov 28;32(4):376–85. doi: 10.1264/jsme2.ME17131 (PMC5745023; doi:10.1264/jsme2.ME17131)
Supplement: Supplementary file 1 [file 32_376_s1.pdf]

Supplemental Table S1. Read number of 16S rRNA gene and ITS region in each sample.

Field name	Sample number	Layer	Read number	
			For prokaryote	For eukaryote
Ha-1	1	Upper	9915	1266
		Lower	10427	1530
	2	Upper	7877	1348
		Lower	8991	NA
	3	Upper	6569	NA
		Lower	9943	1462
	4	Upper	10859	1134
		Lower	9956	2155
	5	Upper	9814	NA
		Lower	10274	4394
Ha-2	1	Upper	6234	NA
		Lower	5992	1680
	2	Upper	5473	1103
		Lower	6322	651
	3	Upper	4715	1576
		Lower	5445	1877
	4	Upper	6252	2015
		Lower	5773	1651
	5	Upper	6763	1425
		Lower	5814	1330
Ka-1	3	Upper	4750	1920
		Lower	5463	1884
	4	Upper	6449	1786
		Lower	5252	1758
	5	Upper	6131	2988
		Lower	6000	3518
Ka-2	1	Upper	14289	4175
		Lower	12138	1373
	2	Upper	12100	1818
		Lower	12097	NA
	3	Upper	9830	2556
		Lower	10225	1034

	4	Upper	10990	2695
		Lower	10283	2187
	5	Upper	10339	2075
		Lower	9598	NA
Wa-1	1	Upper	1668	895
		Lower	2312	1031
	2	Upper	2450	NA
		Lower	1639	1087
	3	Upper	3148	1583
		Lower	7897	NA
	4	Upper	3768	NA
		Lower	7496	NA
Wa-2	3	Upper	10263	1719
		Lower	8643	966
Wa-3	1	Upper	7428	1351
		Lower	6646	1524
	2	Upper	7245	NA
		Lower	6374	1418
	3	Upper	7687	965
		Lower	4150	542
	4	Upper	8442	1183
		Lower	6312	1598

NA represents a PCR amplicon that was not obtained.

Supplemental Table S2. Relative abundance of prokaryotic and eukaryotic phyla in each soil layer of S- and C-soils.

		Ha-field				Ka-field				Wa-field			
	Soil layer	Upper		Lower		Upper		Lower		Upper		Lower	
	Classification	S-soil	C-soil	S-soil	C-soil	S-soil	C-soil	S-soil	C-soil	S-soil	C-soil	S-soil	C-soil
For prokaryote	Proteobacteria	0.32±0.02*	0.28±0.01	0.36±0.03*	0.33±0.01	0.53±0.08	0.42±0.03	0.47±0.07*	0.30±0.08	0.50±0.22	0.30±0.01	0.29±0.07	0.26±0.13
	Firmicutes	0.15±0.02	0.13±0.02	0.13±0.04	0.08±0.04	0.06±0.02	0.14±0.01*	0.05±0.01	0.10±0.04	0.13±0.08	0.10±0.00	0.24±0.1	0.30±0.09
	Acidobacteria	0.09±0.01	0.07±0.0001	0.09±0.02	0.09±0.01	0.12±0.05	0.07±0.01	0.11±0.03	0.31±0.13*	0.03±0.03	0.22±0.00*	0.04±0.01	0.05±0.02
	Bacteroidetes	0.04±0.01	0.08±0.01*	0.04±0.01	0.06±0.03	0.13±0.06	0.13±0.01	0.11±0.04*	0.02±0.01	0.11±0.07	0.04±0.00	0.11±0.05	0.08±0.05
	Chloroflexi	0.11±0.01	0.13±0.01	0.11±0.01	0.11±0.01	0.03±0.02	0.04±0.01	0.03±0.001	0.07±0.03	0.03±0.02	0.05±0.00	0.04±0.01	0.05±0.02
	Actinobacteria	0.04±0.01	0.04±0.0001	0.04±0.01*	0.03±0.01	0.06±0.02	0.04±0.00	0.09±0.02*	0.05±0.02	0.05±0.03	0.04±0.0001	0.12±0.07	0.17±0.12
	Gemmatimonadetes	0.06±0.01	0.06±0.01	0.05±0.01	0.05±0.0001	0.03±0.02	0.04±0.0001	0.02±0.0001	0.02±0.01	0.05±0.03	0.08±0.0001	0.07±0.02	0.05±0.02
	Verrucomicrobia	0.03±0.003	0.03±0.0001	0.01±0.004	0.01±0.01	0.01±0.01	0.05±0.01*	0.01±0.01	0.02±0.01	0.03±0.02	0.03±0.0001	0.03±0.01	0.01±0.003
	Planctomycetes	0.03±0.002	0.05±0.0001*	0.03±0.003	0.04±0.01	0.01±0.01	0.02±0.003*	0.01±0.002	0.02±0.004	0.01±0.01	0.03±0.0001	0.01±0.004	0.01±0.01
	Crenarchaeota	0.01±0.00	0.02±0.0001	0.02±0.01	0.02±0.01	0.001±0.0001	0.03±0.01	0.05±0.05	0.04±0.02	0.003±0.01	0.02±0.0001*	0.002±0.0001	0.003±0.004
	Others	0.11±0.02	0.11±0.03	0.15±0.05	0.18±0.06	0.04±0.02	0.07±0.01	0.05±0.02	0.05±0.02	0.05±0.04	0.01±0.0001	0.04±0.02	0.03±0.01
For eukaryote	Ascomycota	0.25±0.06	0.26±0.11	0.32±0.11	0.39±0.17	0.56±0.12	0.67±0.13	0.53±0.03	0.47±0.21	0.95±0.06	0.99±0.08	0.94±0.01	0.999±0.00
	Basidiomycota	0.04±0.02	0.03±0.01	0.01±0.03	0.006±0.01	0.33±0.12	0.25±0.07	0.24±0.02	0.16±0.05	0.01±0.02	0	0.01±0.0001	0
	Chytridiomycota	0.04±0.02	0.04±0.01	0.02±0.07	0.002±0.003	0.001±0.002	0.003±0.001	0.005±0.004	0.003±0.004	0	0	0.00014±0.00001	0
	Glomeromycota	0.03±0.01	0.43±0.03*	0.03±0.11	0.28±0.08*	0.002±0.001	0.012±0.001*	0.002±0.004	0.01±0.01	0.002±0.002	0.002±0.005	0.003±0.003	0.0005±0.001
	Rozellomycota	0.03±0.03	0.002±0.005	0.005±0.003	0.0002±0.0001	0.001±0.0001	0.002±0.003	0.004±0.002	0.003±0.003	0	0	0.0006±0.0001	0
	Zygomycota	0.32±0.23	0.17±0.06	0.17±0.05	0.21±0.22	0.09±0.05	0.05±0.2	0.2±0.01	0.33±0.2	0.002±0.003	0	0.002±0.0001	0

Cercozoa	0.02±0.0001	0.02±0.01	0.01±0.01	0.02±0.01	0	0.002±0.001	0.001±0.002	0.002±0.001	0	0	0	0
Ciliophora	0.28±0.14*	0.05±0.16	0.45±0.03*	0.09±0.07	0.01±0.0001	0.01±0.0001	0.01±0.0001	0.02±0.02	0.03±0.06	0±0.08	0.04±0.01	0

Asterisk represents a significantly higher value than found in the other soil type in the same soil layer in each field (t-test, $p < 0.05$).

Supplemental Table S3. Prokaryote OTUs that are significantly abundant in S-soil Ha-fields

			Phylum	Class	Closest relatives
Ha-field	All	OTU5287	Acidobacteria	Acidobacteria_Gp1	
		OTU3677	Acidobacteria	Acidobacteria_Gp1	
		OTU15103	Acidobacteria	Acidobacteria_Gp3	
		OTU11151	Acidobacteria	Acidobacteria_Gp3	
		OTU6998	Acidobacteria	Acidobacteria_Gp3	
		OTU24220	Acidobacteria	Acidobacteria_Gp4	
		OTU2090	Acidobacteria	Acidobacteria_Gp6	
		OTU20243	Acidobacteria	Acidobacteria_Gp6	
		OTU19437	Acidobacteria	Acidobacteria_Gp6	
		OTU15871	Acidobacteria	Acidobacteria_Gp7	
		OTU206	Acidobacteria	Acidobacteria_Gp9	
		OTU16664	Acidobacteria	Acidobacteria_Gp11	
		OTU6401	Acidobacteria	Acidobacteria_Gp15	
		OTU17394	Acidobacteria	Acidobacteria_Gp16	
		OTU6911	Acidobacteria	Acidobacteria_Gp16	
		OTU13197	Actinobacteria	Actinobacteria	Thermomonosporaceae
		OTU8401	Actinobacteria	Actinobacteria	Actinocorallia
		OTU22248	Actinobacteria	Actinobacteria	
		OTU1664	Actinobacteria	Actinobacteria	
		OTU5016	Bacteroidetes		
		OTU6552	Bacteroidetes		
		OTU19664	Bacteroidetes	Cytophagia	Cytophagales
		OTU6162	Candidatus Saccharibacteria		
		OTU18561	Candidatus Saccharibacteria		
		OTU9980	Chlamydiae	Chlamydiia	Chlamydiales
		OTU3560	Chlamydiae	Chlamydiia	Chlamydiales
		OTU15407	Chlamydiae	Chlamydiia	Parachlamydiaceae
		OTU16695	Chloroflexi	Anaerolineae	Anaerolineaceae
		OTU9792	Chloroflexi	Anaerolineae	Anaerolineaceae
		OTU2182	Chloroflexi	Anaerolineae	Anaerolineaceae
		OTU3967	Chloroflexi	Anaerolineae	Anaerolineaceae
		OTU17184	Chloroflexi	Caldilineae	Litorilinea

OTU5397	Chloroflexi		
OTU7397	Firmicutes	Bacilli	Bacillales
OTU23176	Firmicutes	Bacilli	Paenibacillaceae
OTU3667	Firmicutes	Clostridia	Clostridiaceae
OTU21676	Firmicutes	Clostridia	Clostridiales
OTU11100	Firmicutes	Clostridia	Clostridium
OTU11671	Firmicutes	Clostridia	Clostridiales
OTU2261	Firmicutes	Clostridia	Clostridiaceae
OTU7053	Firmicutes	Clostridia	Clostridium
OTU10441	Firmicutes	Negativicutes	Veillonellaceae
OTU16279	Gemmatimonadetes	Gemmatimonadetes	Gemmatimonas
OTU21491	Ignavibacteriae	Ignavibacteria	Ignavibacterium
OTU23542	Microgenomates		
OTU6594	Nitrospirae	Nitrospira	Nitrospira
OTU23315	Pacearchaeota	Pacearchaeota	
OTU7118	Pacearchaeota	Pacearchaeota	
OTU801	Parcubacteria		
OTU18319	Parcubacteria		
OTU7382	Planctomycetes	Planctomycetia	
OTU12618	Planctomycetes	Planctomycetia	
OTU9025	Planctomycetes	Planctomycetia	Planctomycetaceae
OTU17862	Planctomycetes	Planctomycetia	Pirellula
OTU14848	Proteobacteria	Alphaproteobacteria	Rhizobiales
OTU20963	Proteobacteria	Alphaproteobacteria	Rhizobiales
OTU568	Proteobacteria	Alphaproteobacteria	Rhizobiales
OTU389	Proteobacteria	Alphaproteobacteria	Rhizobiales
OTU22478	Proteobacteria	Betaproteobacteria	Tepidiphilus
OTU15570	Proteobacteria	Betaproteobacteria	
OTU17694	Proteobacteria	Betaproteobacteria	Noviherbaspirillum
OTU6510	Proteobacteria	Betaproteobacteria	
OTU20479	Proteobacteria	Deltaproteobacteria	Deltaproteobacteria
OTU2441	Proteobacteria	Deltaproteobacteria	Nannocystineae
OTU8435	Proteobacteria	Deltaproteobacteria	
OTU24632	Proteobacteria	Deltaproteobacteria	Geobacter
OTU15844	Proteobacteria	Gammaproteobacteria	Legionella
OTU15518	Proteobacteria	Gammaproteobacteria	Chromatiales

	OTU20900	Proteobacteria	Gammaproteobacteria	
	OTU550	Proteobacteria	Gammaproteobacteria	
	OTU7618	Proteobacteria	Gammaproteobacteria	
	OTU2709	Proteobacteria		
	OTU9673	Proteobacteria		
	OTU1614	Proteobacteria		
	OTU8827	Proteobacteria		
	OTU7150	Proteobacteria		
	OTU12647	Verrucomicrobia	Subdivision3	
	OTU1990	Verrucomicrobia	Subdivision3	
Upper	OTU9379	Acidobacteria	Acidobacteria_Gp5	
	OTU8201	Acidobacteria	Acidobacteria_Gp3	
	OTU13494	Acidobacteria	Acidobacteria_Gp1	
	OTU12100	Actinobacteria	Actinobacteria	Actinomycetales
	OTU9174	Actinobacteria	Actinobacteria	
	OTU12247	Armatimonadetes	Armatimonadetes_gp2	
	OTU24568	Bacteroidetes	Sphingobacteriia	Pedobacter
	OTU18176	Bacteroidetes	Sphingobacteriia	Sphingobacteriaceae
	OTU11194	Bacteroidetes		
	OTU3438	Bacteroidetes		
	OTU21855	Bacteroidetes	Sphingobacteriia	Chitinophagaceae
	OTU8957	Chlamydiae	Chlamydiia	Chlamydiales
	OTU19508	Chlamydiae	Chlamydiia	Simkania
	OTU2696	Chlamydiae	Chlamydiia	Chlamydiales
	OTU21888	Chloroflexi	Anaerolineae	Anaerolineaceae
	OTU3264	Chloroflexi	Anaerolineae	Anaerolineaceae
	OTU15846	Firmicutes	Bacilli	Thermoactinomycetaceae
	OTU21315	Firmicutes	Bacilli	Bacillaceae
	OTU4197	Firmicutes	Bacilli	Bacillaceae
	OTU6267	Firmicutes	Bacilli	Thermoactinomycetaceae
	OTU6709	Firmicutes	Bacilli	Bacillaceae
	OTU4149	Firmicutes	Clostridia	Clostridiaceae
	OTU225	Firmicutes	Clostridia	Ruminococcaceae
	OTU20230	Firmicutes	Clostridia	Clostridiales
	OTU21927	Firmicutes	Negativicutes	Pelosinus
	OTU9676	Gemmatimonadetes	Gemmatimonadetes	Gemmatimonas

	OTU16633	Ignavibacteriae	Ignavibacteria	Ignavibacteriaceae
	OTU23472	Pacearchaeota	Pacearchaeota	
	OTU6487	Parcubacteria	Parcubacteria_genera	
	OTU3269	Planctomycetes	Planctomycetia	Planctomycetaceae
	OTU899	Planctomycetes	Planctomycetia	Planctomycetaceae
	OTU19765	Planctomycetes	Planctomycetia	Blastopirellula
	OTU19052	Proteobacteria	Alphaproteobacteria	
	OTU5978	Proteobacteria	Alphaproteobacteria	Rhodobiaceae
	OTU4243	Proteobacteria	Alphaproteobacteria	Acetobacteraceae
	OTU22130	Proteobacteria	Alphaproteobacteria	Microvirga
	OTU13774	Proteobacteria	Alphaproteobacteria	Rhizobiales
	OTU6310	Proteobacteria	Betaproteobacteria	Thauera
	OTU1523	Proteobacteria	Deltaproteobacteria	Desulfatiglans
	OTU180	Proteobacteria	Deltaproteobacteria	Nannocystineae
	OTU16960	Proteobacteria	Deltaproteobacteria	Nannocystaceae
	OTU17470	Proteobacteria	Deltaproteobacteria	Cystobacterineae
	OTU22487	Proteobacteria	Deltaproteobacteria	
	OTU8874	Proteobacteria	Deltaproteobacteria	Bdellovibrio
	OTU22496	Proteobacteria	Deltaproteobacteria	Sorangiineae
	OTU21244	Proteobacteria	Deltaproteobacteria	Myxococcales
	OTU12199	Proteobacteria	Deltaproteobacteria	
	OTU1711	Proteobacteria	Gammaproteobacteria	
	OTU14295	Proteobacteria	Gammaproteobacteria	Chiayiivirga
	OTU15847	Proteobacteria	Gammaproteobacteria	Aquicella
	OTU11531	Proteobacteria	Gammaproteobacteria	
	OTU16634	Proteobacteria	Gammaproteobacteria	Xanthomonadales
	OTU14364	Proteobacteria		
	OTU6366	Verrucomicrobia	Verrucomicrobiae	Luteolibacter
	OTU17098	Woesearchaeota	Woesearchaeota	
Lower	OTU21177	Acidobacteria	Acidobacteria_Gp1	
	OTU15025	Acidobacteria	Acidobacteria_Gp3	
	OTU15833	Acidobacteria	Acidobacteria_Gp4	Acidobacteria_Gp4
	OTU16307	Acidobacteria	Acidobacteria_Gp4	
	OTU9926	Acidobacteria	Acidobacteria_Gp6	
	OTU12648	Acidobacteria	Acidobacteria_Gp15	
	OTU5651	Acidobacteria	Acidobacteria_Gp17	

OTU23424	Acidobacteria	Acidobacteria_Gp25	
OTU5584	Acidobacteria		
OTU302	Acidobacteria		
OTU15266	Actinobacteria	Actinobacteria	Actinomadura
OTU9194	Actinobacteria	Actinobacteria	Conexibacter
OTU17676	Actinobacteria	Actinobacteria	Gaiella
OTU10679	Actinobacteria	Actinobacteria	Conexibacter
OTU4477	Actinobacteria	Actinobacteria	
OTU22937	Armatimonadetes	Armatimonadetes Gp4	
OTU7268	Bacteroidetes	Sphingobacteriia	Chitinophagaceae
OTU18740	Bacteroidetes	Sphingobacteriia	Sphingobacteriales
OTU20426	Bacteroidetes		
OTU1933	Bacteroidetes	Flavobacteriia	Flavobacterium
OTU12667	candidate division WPS-1		
OTU21056	Chlamydiae	Chlamydiia	Chlamydiales
OTU22836	Chloroflexi	Caldilineae	Caldilineaceae
OTU4251	Chloroflexi	Anaerolineae	Anaerolineaceae
OTU12324	Euryarchaeota		
OTU12031	Firmicutes	Clostridia	Peptococcaceae
OTU20927	Firmicutes	Clostridia	Clostridiaceae
OTU2723	Firmicutes	Negativicutes	Veillonellaceae
OTU12442	Firmicutes		
OTU23312	Gemmatimonadetes	Gemmatimonadetes	Gemmatimonas
OTU24825	Gemmatimonadetes	Gemmatimonadetes	Gemmatimonas
OTU20399	Hydrogenedentes	Candidatus	
OTU11530	Microgenomates		
OTU3554	Pacearchaeota	Pacearchaeota	
OTU5090	Pacearchaeota	Pacearchaeota	
OTU1314	Parcubacteria		
OTU22801	Planctomycetes	Planctomycetia	Planctomycetaceae
OTU13582	Planctomycetes	Planctomycetia	Planctomycetaceae
OTU22015	Planctomycetes	Planctomycetia	Planctomycetaceae
OTU13634	Proteobacteria	Alphaproteobacteria	
OTU16131	Proteobacteria	Alphaproteobacteria	Rhizobiales
OTU13395	Proteobacteria	Alphaproteobacteria	
OTU11020	Proteobacteria	Alphaproteobacteria	

		OTU3591	Proteobacteria	Alphaproteobacteria	Rhodospirillaceae
		OTU23957	Proteobacteria	Betaproteobacteria	
		OTU9079	Proteobacteria	Betaproteobacteria	
		OTU24488	Proteobacteria	Betaproteobacteria	Nitrosomonas
		OTU7518	Proteobacteria	Deltaproteobacteria	Nannocystineae
		OTU19764	Proteobacteria	Deltaproteobacteria	Myxococcales
		OTU17939	Proteobacteria	Deltaproteobacteria	Nannocystineae
		OTU335	Proteobacteria	Deltaproteobacteria	Geobacter
		OTU14882	Proteobacteria	Deltaproteobacteria	
		OTU18221	Proteobacteria	Deltaproteobacteria	Desulfuromonadaceae
		OTU20209	Proteobacteria	Gammaproteobacteria	Chromatiales
		OTU3632	Proteobacteria	Gammaproteobacteria	Sinobacteraceae
		OTU5210	Proteobacteria	Gammaproteobacteria	
		OTU7792	Proteobacteria	Gammaproteobacteria	
		OTU2751	Proteobacteria	Gammaproteobacteria	
		OTU4462	Proteobacteria	Gammaproteobacteria	
		OTU15474	Proteobacteria	Gammaproteobacteria	
		OTU6008	Proteobacteria	Gammaproteobacteria	
		OTU14766	Proteobacteria		
		OTU24831	Proteobacteria		
		OTU13607	Proteobacteria		
		OTU17948	Verrucomicrobia	Subdivision3	
		OTU21712	Verrucomicrobia		
Ka-field	All	OTU18265	Acidobacteria	Acidobacteria_Gp1	
		OTU24883	Acidobacteria	Acidobacteria_Gp1	
		OTU15236	Acidobacteria	Acidobacteria_Gp2	
		OTU2494	Acidobacteria	Acidobacteria_Gp2	
		OTU2904	Acidobacteria	Acidobacteria_Gp3	
		OTU10890	Acidobacteria	Acidobacteria_Gp3	
		OTU3814	Acidobacteria	Acidobacteria_Gp3	
		OTU10306	Acidobacteria	Acidobacteria_Gp6	
		OTU15016	Acidobacteria	Acidobacteria_Gp6	
		OTU10267	Acidobacteria	Acidobacteria_Gp13	
		OTU18813	Acidobacteria	Acidobacteria_Gp14	
		OTU10061	Actinobacteria	Actinobacteria	Catenulispora
		OTU958	Actinobacteria	Actinobacteria	Micromonosporaceae

OTU1804	Actinobacteria	Actinobacteria	Corynebacterium
OTU8187	Actinobacteria	Actinobacteria	Acidimicrobineae
OTU298	Actinobacteria	Actinobacteria	Gaiella
OTU11978	Actinobacteria	Actinobacteria	Enteractinococcus
OTU24612	Actinobacteria	Actinobacteria	Conexibacter
OTU2575	Actinobacteria	Actinobacteria	Solirubrobacterales
OTU16877	Actinobacteria	Actinobacteria	Streptosporangineae
OTU13324	Actinobacteria	Actinobacteria	Acidimicrobineae
OTU10838	Actinobacteria	Actinobacteria	Aciditerrimonas
OTU7753	Bacteroidetes	Cytophagia	Algoriphagus
OTU8729	Bacteroidetes	Cytophagia	Flammeovirgaceae
OTU4616	Bacteroidetes	Cytophagia	Cytophagales
OTU3223	Bacteroidetes	Flavobacteriia	Flavobacterium
OTU23347	Bacteroidetes	Flavobacteriia	Flavobacteriaceae
OTU24132	Bacteroidetes	Sphingobacteriia	Chitinophagaceae
OTU16566	Bacteroidetes	Sphingobacteriia	Chitinophagaceae
OTU23672	Bacteroidetes	Sphingobacteriia	Chitinophagaceae
OTU5106	Bacteroidetes	Sphingobacteriia	Chitinophagaceae
OTU23129	Bacteroidetes	Sphingobacteriia	Pedobacter
OTU6371	Bacteroidetes	Sphingobacteriia	Chitinophaga
OTU14796	Bacteroidetes	Sphingobacteriia	Chitinophagaceae
OTU22561	Bacteroidetes	Sphingobacteriia	Arcticibacter
OTU24657	Bacteroidetes	Sphingobacteriia	Taibaiella
OTU10882	Bacteroidetes	Sphingobacteriia	Pedobacter
OTU12664	Bacteroidetes		
OTU2467	Firmicutes	Bacilli	Bacillales
OTU18934	Firmicutes	Bacilli	Paenibacillaceae
OTU11864	Firmicutes	Bacilli	
OTU7184	Firmicutes	Bacilli	Lactobacillus
OTU16493	Firmicutes	Bacilli	Paenibacillaceae
OTU18373	Firmicutes	Clostridia	Clostridiales_Incertae
OTU20291	Firmicutes		
OTU24237	Planctomycetes	Planctomycetia	Planctomycetaceae
OTU18726	Proteobacteria	Alphaproteobacteria	Acetobacteraceae
OTU6002	Proteobacteria	Alphaproteobacteria	Rhizomicrobium
OTU1767	Proteobacteria	Alphaproteobacteria	Sphingomonadaceae

	OTU11271	Proteobacteria	Alphaproteobacteria	Sphingomonadaceae
	OTU22016	Proteobacteria	Alphaproteobacteria	Rhizobiales
	OTU15766	Proteobacteria	Alphaproteobacteria	
	OTU18787	Proteobacteria	Betaproteobacteria	Burkholderiales
	OTU9437	Proteobacteria	Deltaproteobacteria	Myxococcales
	OTU15551	Proteobacteria	Deltaproteobacteria	Polyangiaceae
	OTU15693	Proteobacteria	Gammaproteobacteria	
	OTU1194	Proteobacteria	Gammaproteobacteria	
	OTU23135	Proteobacteria	Gammaproteobacteria	
	OTU15450	Proteobacteria	Gammaproteobacteria	
	OTU4736	Proteobacteria	Gammaproteobacteria	Mizugakiibacter
	OTU9171	Proteobacteria	Gammaproteobacteria	
	OTU14098	Proteobacteria	Gammaproteobacteria	Xanthomonadaceae
	OTU7380	Proteobacteria	Gammaproteobacteria	
	OTU16560	Proteobacteria	Gammaproteobacteria	
	OTU19578	Proteobacteria	Gammaproteobacteria	Pseudohongiella
	OTU6252	Proteobacteria	Gammaproteobacteria	Xanthomonadaceae
	OTU10808	Proteobacteria		
	OTU20611	Verrucomicrobia	Spartobacteria	
	OTU626	Verrucomicrobia	Opitutae	Opitutus
Upper	OTU12966	Acidobacteria	Acidobacteria_Gp1	
	OTU4732	Acidobacteria	Acidobacteria_Gp2	
	OTU18537	Actinobacteria	Actinobacteria	Actinomycetales
	OTU1540	Bacteroidetes	Sphingobacteriia	Mucilaginibacter
	OTU3294	Bacteroidetes	Flavobacteriia	Cryomorphaceae
	OTU400	Bacteroidetes		
	OTU5161	Proteobacteria	Alphaproteobacteria	
	OTU20179	Proteobacteria	Alphaproteobacteria	Bradyrhizobiaceae
	OTU1172	Proteobacteria	Alphaproteobacteria	
	OTU20137	Proteobacteria	Alphaproteobacteria	
	OTU21030	Proteobacteria	Alphaproteobacteria	
	OTU21727	Proteobacteria	Alphaproteobacteria	Rhizorhabdus
	OTU11717	Proteobacteria	Alphaproteobacteria	Sphingomonadaceae
	OTU23326	Proteobacteria	Gammaproteobacteria	
	OTU288	Proteobacteria	Gammaproteobacteria	
	OTU12498	Proteobacteria		

		OTU4850	Spirochaetes	Spirochaetia	Spirochaetaceae
		OTU19105	Thaumarchaeota		
	Lower	OTU15729	Actinobacteria	Actinobacteria	Streptacidiphilus
		OTU11592	Actinobacteria	Actinobacteria	Gaiella
		OTU6124	Bacteroidetes	Sphingobacteriia	Chitinophagaceae
		OTU1070	Bacteroidetes	Cytophagia	Cytophaga
		OTU10604	Bacteroidetes	Cytophagia	Chryseolinea
		OTU1553	Bacteroidetes	Sphingobacteriia	Chitinophagaceae
		OTU17890	Bacteroidetes	Flavobacteriia	Wandonia
		OTU830	Bacteroidetes	Sphingobacteriia	Chitinophagaceae
		OTU7262	Chloroflexi	Ktedonobacteria	Ktedonobacterales
		OTU4348	Cyanobacteria/Chloroplast	Chloroplast	Chlorophyta
		OTU15283	Firmicutes	Clostridia	Clostridiaceae
		OTU5896	Firmicutes	Bacilli	Bacillaceae
		OTU11659	Firmicutes	Bacilli	Paenibacillaceae
		OTU7769	Firmicutes	Bacilli	Bacillaceae
		OTU12926	Gemmatimonadetes	Gemmatimonadetes	Gemmatimonas
		OTU417	Nitrospirae	Nitrospira	Nitrospira
		OTU7850	Proteobacteria	Gammaproteobacteria	Xanthomonadaceae
		OTU23106	Proteobacteria	Alphaproteobacteria	Acidisoma
		OTU304	Proteobacteria	Gammaproteobacteria	Povalibacter
		OTU11208	Proteobacteria	Gammaproteobacteria	
		OTU24597	Proteobacteria	Deltaproteobacteria	
		OTU3699	Thaumarchaeota	Nitrososphaerales	Nitrososphaera
Wa-field	All	OTU656	Acidobacteria	Acidobacteria_Gp4	
		OTU15670	Acidobacteria	Acidobacteria_Gp4	
		OTU9831	Actinobacteria	Actinobacteria	Solirubrobacterales
		OTU14617	Actinobacteria	Actinobacteria	Micromonosporaceae
		OTU11752	Bacteroidetes	Cytophagia	Dyadobacter
		OTU7472	Bacteroidetes	Cytophagia	Cytophagales
		OTU19817	Bacteroidetes	Cytophagia	Cytophagales
		OTU17576	Bacteroidetes	Cytophagia	Ohtaekwangia
		OTU21984	Bacteroidetes	Cytophagia	Cytophagales
		OTU6037	Bacteroidetes	Cytophagia	Cytophagales
		OTU93	Bacteroidetes	Sphingobacteriia	Parasegetibacter
		OTU8217	Bacteroidetes	Sphingobacteriia	Sphingobacteriaceae

	OTU4259	Bacteroidetes	Sphingobacteriia	Sphingobacterium
	OTU16975	Bacteroidetes	Sphingobacteriia	Pedobacter
	OTU19866	Bacteroidetes	Sphingobacteriia	Chitinophagaceae
	OTU6973	Bacteroidetes	Sphingobacteriia	Pedobacter
	OTU17302	Bacteroidetes	Sphingobacteriia	Chitinophaga
	OTU9830	Bacteroidetes		
	OTU21611	Bacteroidetes		
	OTU14600	Bacteroidetes		
	OTU11170	Firmicutes	Bacilli	Bacillaceae
	OTU7441	Firmicutes	Bacilli	Paenibacillaceae
	OTU15277	Gemmatimonadetes	Gemmatimonadetes	Gemmatimonas
	OTU3522	Gemmatimonadetes	Gemmatimonadetes	Gemmatimonas
	OTU20374	Proteobacteria	Alphaproteobacteria	Sphingobium
	OTU6089	Proteobacteria	Alphaproteobacteria	Rhizobiales
	OTU7735	Proteobacteria	Alphaproteobacteria	Reyranella
	OTU6138	Proteobacteria	Alphaproteobacteria	Rhodospirillales
	OTU23387	Proteobacteria	Alphaproteobacteria	Geminicoccus
	OTU8038	Proteobacteria	Betaproteobacteria	Methylophilaceae
	OTU20331	Proteobacteria	Betaproteobacteria	Pseudoduganella
	OTU3074	Proteobacteria	Betaproteobacteria	
	OTU16038	Proteobacteria	Gammaproteobacteria	
	OTU1729	Proteobacteria	Gammaproteobacteria	Sinobacteraceae
	OTU18771	Proteobacteria	Gammaproteobacteria	
	OTU23744	Proteobacteria	Gammaproteobacteria	Arenimonas
	OTU962	Proteobacteria		
	OTU15385	Verrucomicrobia	Subdivision3	
	OTU12833	Verrucomicrobia	Subdivision3	
	OTU19086	Verrucomicrobia	Verrucomicrobiae	Prosthecobacter
	OTU20061	Verrucomicrobia	Subdivision3	
Upper	OTU22448	Armatimonadetes	Armatimonadetes_gp5	Armatimonadetes_gp5
	OTU10941	Bacteroidetes	Sphingobacteriia	Chitinophagaceae
	OTU14892	Bacteroidetes	Sphingobacteriia	Chitinophagaceae
	OTU11976	Bacteroidetes	Sphingobacteriia	Chitinophagaceae
	OTU10248	Chlamydiae	Chlamydiia	Simkania
	OTU7264	Chloroflexi	Ktedonobacteria	Thermogemmatispora
	OTU1960	Firmicutes	Clostridia	Peptococcaceae

	OTU22220	Firmicutes	Bacilli	Bacillaceae
	OTU6598	Proteobacteria	Alphaproteobacteria	Pseudochrobactrum
	OTU19958	Proteobacteria	Alphaproteobacteria	Phenylobacterium
	OTU20604	Proteobacteria	Betaproteobacteria	Azoarcus
	OTU9154	Proteobacteria	Deltaproteobacteria	Myxococcales
	OTU10946	Proteobacteria	Gammaproteobacteria	
	OTU22910	Verrucomicrobia	Subdivision3	
	OTU17908	Verrucomicrobia	Opitutae	Opitutus
Lower	OTU10928	Acidobacteria	Acidobacteria_Gp3	
	OTU12071	Acidobacteria	Acidobacteria_Gp6	
	OTU3712	Actinobacteria	Actinobacteria	Actinoallomurus
	OTU13102	Actinobacteria	Actinobacteria	Micromonosporaceae
	OTU4287	Bacteroidetes	Cytophagia	Algoriphagus
	OTU1695	Bacteroidetes	Sphingobacteriia	Chitinophagaceae
	OTU8106	Bacteroidetes	Sphingobacteriia	Terrimonas
	OTU18038	Bacteroidetes		
	OTU6671	Chloroflexi	Thermomicrobia	Nitrolancea
	OTU22036	Deinococcus-Thermus	Deinococci	Meiothermus
	OTU7785	Firmicutes	Bacilli	Bacillaceae
	OTU23960	Firmicutes	Bacilli	Thermoactinomycetaceae
	OTU20290	Gemmatimonadetes	Gemmatimonadetes	Gemmatimonas
	OTU9952	Gemmatimonadetes	Gemmatimonadetes	Gemmatimonas
	OTU1772	Planctomycetes	Planctomycetia	Planctomycetaceae
	OTU23249	Planctomycetes	Planctomycetia	Planctomycetaceae
	OTU17127	Proteobacteria	Alphaproteobacteria	Rhizobiales
	OTU21082	Proteobacteria	Deltaproteobacteria	Myxococcales
	OTU6350	Proteobacteria		
	OTU23827	Proteobacteria	Betaproteobacteria	

Supplemental Table S4. Prokaryote OTUs that are significantly abundant in S-soil Ha-fields

		Name			
Ha-field	All	OTU260	Fungi	Ascomycota	Rasamsonia brevistipitata strain CBS 128786
		OTU410	Fungi	Ascomycota	Pochonia sp. isolate TTC169
		OTU1856	Fungi	Ascomycota	Geomyces sp. 23WI14
		OTU725	Fungi	Ascomycota	Geomyces sp. 23WI14
		OTU326	Fungi	Basidiomycota	Conocybe aff. ochrostriata NL-0830
		OTU283	Viridiplantae	Chlorophyta	Protosiphon botryoides
		OTU238	Viridiplantae	Chlorophyta	Chlorococcum sp. CCAP 11/52
		OTU576	Viridiplantae	Chlorophyta	Chlorella pyrenoidosa isolate 2_1
		OTU411	Viridiplantae	Chlorophyta	Chlamydomonas sp. CCAP
		OTU164	Alveolata	Ciliophora	Oxytricha longigranulosa macronuclear
		OTU201	Alveolata	Ciliophora	Apoholosticha sinica voucher PY2011041303
		OTU589	Alveolata	Ciliophora	Oxytricha longigranulosa macronuclear
		OTU113	Alveolata	Ciliophora	Oxytricha longigranulosa macronuclear
		OTU2140	Alveolata	Ciliophora	Homalogastra setosa isolate GT1 clone B
		OTU199	Nematoda	Adenophorea	Pratylenchus goodeyi isolate PgoKL5 clone 5
		OTU1665	Viridiplantae	Streptophyta	Imbribryum blandum
		OTU313	Viridiplantae	Streptophyta	Imbribryum blandum
		OTU304	Viridiplantae	Streptophyta	Imbribryum blandum
		OTU53	Viridiplantae	Streptophyta	Imbribryum blandum
		OTU99	Viridiplantae	Streptophyta	Imbribryum blandum
		OTU754	Viridiplantae	Streptophyta	Imbribryum blandum
	Up	OTU1325	Fungi	Basidiomycota	Psathyrella kellermanii voucher de Meulder11242
		OTU966	Fungi	Mucoromycota	Mortierella oligospora isolate SDMt
		OTU262	Fungi	Unidentified Fungi	Fungal endophyte isolate 07
	Low	OTU625	Fungi	Ascomycota	Halosphaeriaceae sp.
		OTU964	Fungi	Ascomycota	Veronaea sp. E6917h
		OTU952	Fungi	Ascomycota	Zopfiella lundqvistii
		OTU392	Fungi	Ascomycota	Ascomycota sp. UNEX FECRGA 2012E703
		OTU696	Fungi	Ascomycota	Cercophora sparsa voucher
		OTU9762	Fungi	Ascomycota	Scutellinia sp. OTU101 AN-2016
		OTU587	Fungi	Ascomycota	Penicillium sp. KRP51

OTU697	Fungi	Ascomycota	Fusicolla acetilerea isolate 248WS
OTU388	Fungi	Ascomycota	Fusarium sp. strain Zbf-S1
OTU1766	Fungi	Ascomycota	Geomyces sp. 23WI14
OTU850	Fungi	Ascomycota	Debaryomyces maramus culture-collection CBS:1958
OTU1601	Fungi	Ascomycota	Coniochaetales sp. GMG_C4
OTU637	Fungi	Ascomycota	Acremonium alternatum
OTU1263	Fungi	Ascomycota	Ascomycota sp. strain SA340
OTU705	Fungi	Ascomycota	Monascus purpureus clone B41-06
OTU174	Fungi	Ascomycota	Acremonium sp. S5-T-2-8
OTU871	Fungi	Ascomycota	Humicola grisea var. grisea
OTU965	Fungi	Ascomycota	Zopfiella sp. TMS-2011 voucher BGd10p15-14
OTU1896	Fungi	Basidiomycota	Psathyrella orbicularis voucher LO149-11
OTU528	Fungi	Basidiomycota	Leucoagaricus birnbaumii
OTU472	Fungi	Mucoromycota	Mortierella alpina
OTU1981	Fungi	Unidentified Fungi	Fungal sp. strain Sy8
OTU815	Rhizaria	Cercozoa	Thaumatomonas sp. P102
OTU701	Rhizaria	Cercozoa	Allas aff. diplophysa
OTU673	Rhizaria	Cercozoa	Thaumatomonas sp. 6 JMS-2012
OTU81	Alveolata	Ciliophora	Epispathidium sp. RV-2015
OTU1727	Alveolata	Ciliophora	Stylonychia lemnae
OTU1895	Alveolata	Ciliophora	Stylonychia lemnae
OTU279	Alveolata	Ciliophora	Stylonychia lemnae
OTU207	Alveolata	Ciliophora	Paracladotricha salina voucher 0605232C
OTU119	Alveolata	Ciliophora	Apoholosticha sinica voucher PY2011041303
OTU737	Alveolata	Ciliophora	Oxytricha longigranulosa
OTU2118	Alveolata	Ciliophora	Paracladotricha salina voucher 0605232C
OTU1764	Alveolata	Ciliophora	Apoholosticha sinica voucher PY2011041303
OTU1745	Alveolata	Ciliophora	Paracladotricha salina voucher 0605232C
OTU804	Alveolata	Ciliophora	Tachysoma pellionellum voucher JJM09022601
OTU1556	Alveolata	Ciliophora	Apoholosticha sinica voucher PY2011041303
OTU2124	Alveolata	Ciliophora	Paracladotricha salina voucher 0605232C
OTU270	Alveolata	Ciliophora	Oxytricha longigranulosa macronuclear
OTU429	Alveolata	Ciliophora	Sterkiella histriomuscorum
OTU810	Nematoda	Adenophorea	Pratylenchus sp. Autumn75
OTU2197	Viridiplantae	Chlorophyta	Tetracystis tetraspora strain SAG 98.80

		OTU633	Viridiplantae	Chlorophyta	Pseudostichococcus monallantoides strain SAG 380-1
		OTU515	Viridiplantae	Chlorophyta	Chlamydomonadales sp. LBA 40
		OTU1015	Viridiplantae	Chlorophyta	Protosiphon botryoides
		OTU513	Viridiplantae	Chlorophyta	Chlorochytrium sp. SAG 212-1
		OTU1321	Viridiplantae	Chlorophyta	Chlorochytrium sp. SAG 212-1
		OTU416	Viridiplantae	Chlorophyta	Chlorochytrium sp. SAG 212-1
		OTU957	Viridiplantae	Streptophyta	Imbribryum blandum
		OTU2021	Viridiplantae	Streptophyta	Imbribryum blandum
		OTU314	Viridiplantae	Streptophyta	Imbribryum blandum
		OTU851	Viridiplantae	Streptophyta	Imbribryum blandum
		OTU582	Viridiplantae	Streptophyta	Imbribryum blandum
		OTU1544	Viridiplantae	Streptophyta	Imbribryum blandum
		OTU1681	Viridiplantae	Streptophyta	Imbribryum blandum
		OTU547	Viridiplantae	Streptophyta	Imbribryum blandum
Ka-field	All	OTU413	Fungi	Ascomycota	Cladophialophora sp. KO-groupI 2014
		OTU236	Fungi	Ascomycota	Fusarium sp. R194
		OTU419	Fungi	Ascomycota	Volutella ciliata strain WBS025
		OTU730	Fungi	Ascomycota	Pleosporales sp. KO-groupE 2014
		OTU63	Fungi	Ascomycota	Sordariomycetes sp. genotype 576 isolate NC1010
		OTU1291	Fungi	Ascomycota	Stagonosporopsis cucurbitacearum strain HGGJ-7
		OTU84	Fungi	Ascomycota	Myceliophthora verrucosa
		OTU144	Fungi	Ascomycota	Sarcopodium circinosetiferum isolate PSN02
		OTU588	Fungi	Basidiomycota	Apiotrichum porosum strain A1C(3)
		OTU1515	Fungi	Basidiomycota	Trichosporon sp. 'shinodae' culture-collection CBS:9979
		OTU1757	Fungi	Basidiomycota	Trichosporon sp. 'shinodae' culture-collection CBS:9979
		OTU1294	Fungi	Basidiomycota	Trichosporon sp. 'shinodae' culture-collection CBS:9979
		OTU424	Fungi	Basidiomycota	Apiotrichum porosum strain A1C(3)
		OTU155	Fungi	Basidiomycota	Apiotrichum porosum strain A1C(3)
		OTU184	Fungi	Basidiomycota	Apiotrichum dulcitum culture-collection CBS:8260
		OTU386	Fungi	Basidiomycota	Apiotrichum porosum strain A1C(3)
		OTU982	Fungi	Basidiomycota	Basidiomycota sp. CC 15-08
		OTU484	Fungi	Basidiomycota	Leucosporidium creatinivorum strain DBVPG 5866
		OTU80	Fungi	Basidiomycota	Conocybe intrusa strain WU25546
		OTU1431	Fungi	Mucoromycota	Umbelopsis sp. nwa_besc_211ck
		OTU263	Fungi	Mucoromycota	Diversisporales sp. isolate CL0057_49_18
		OTU189	Fungi	Mucoromycota	Isomucor trufemiae strain CCIBt 2328

		OTU393	Fungi	Mucoromycota	Mortierella hyalina strain FSU 1954
		OTU135	Fungi	Mucoromycota	Isomucor trufemiae strain CCIBt 2328
		OTU579	Viridiplantae	Chlorophyta	Bracteacoccus minor strain KF 5
		OTU216	Viridiplantae	Chlorophyta	Chlorella vulgaris strain KNUA027
		OTU123	Fungi	Zoopagomycota	Syncephalis obconica
	Up	OTU978	Fungi	Ascomycota	Trichocladium sp. OTU052 AN-2016
		OTU1500	Fungi	Ascomycota	Helicoma sp. NRRL 66050
		OTU1955	Fungi	Basidiomycota	Trichosporon sp. 'shinodae' culture-collection CBS:9979
		OTU511	Fungi	Mucoromycota	Umbelopsis sp. nwa_besc_211ck
		OTU2074	Fungi	Unidentified Fungi	Fungal sp. AM2013 strain 205_Rm i
		OTU233	Alveolata	Ciliophora	Oxytricha longa
	Low	OTU451	Fungi	Ascomycota	Pseudogymnoascus sp. isolate TTC448
		OTU1905	Fungi	Ascomycota	Sordariomycetes sp. OGPBioA15
		OTU1481	Fungi	Basidiomycota	Basidiomycota sp. CC 15-08
		OTU433	Fungi	Basidiomycota	Rhodotorula kratochvilovae culture-collection CBS:5992
		OTU1447	Fungi	Mucoromycota	Umbelopsis vinacea isolate PDW2-5
		OTU1462	Fungi	Unidentified Fungi	Fungal endophyte strain C384T
Wa-field	All	OTU376	Fungi	Ascomycota	Plectosphaerella cucumerina
		OTU176	Fungi	Ascomycota	Penicillium mexicanum CBS 138227
	Up	OTU183	Fungi	Basidiomycota	Clitopilus scyphoides isolate T-777
	Low	OTU312	Fungi	Ascomycota	Zopfiella sp. G367 i
		OTU979	Fungi	Ascomycota	Dothideomycetes sp. XJGY-6
		OTU707	Fungi	Ascomycota	Candida sp. G1
